# Resurrecting extinct cephalopods with biomimetic robots to explore hydrodynamic stability, maneuverability, and physical constraints on life habits

**DOI:** 10.1038/s41598-022-13006-6

**Published:** 2022-07-04

**Authors:** David J. Peterman, Kathleen A. Ritterbush

**Affiliations:** Department of Geology and Geophysics, University of Utah, Salt Lake City, UT USA

**Keywords:** Palaeoecology, Palaeontology

## Abstract

Externally shelled cephalopods with coiled, planispiral conchs were ecologically successful for hundreds of millions of years. These animals displayed remarkable morphological disparity, reflecting comparable differences in physical properties that would have constrained their life habits and ecological roles. To investigate these constraints, self-propelling, neutrally buoyant, biomimetic robots were 3D-printed for four disparate morphologies. These robots were engineered to assume orientations computed from virtual hydrostatic simulations while producing *Nautilus*-like thrusts. Compressed morphotypes had improved hydrodynamic stability (coasting efficiency) and experienced lower drag while jetting backwards. However, inflated morphotypes had improved maneuverability while rotating about the vertical axis. These differences highlight an inescapable physical tradeoff between hydrodynamic stability and yaw maneuverability, illuminating different functional advantages and life-habit constraints across the cephalopod morphospace. This tradeoff reveals there is no single optimum conch morphology, and elucidates the success and iterative evolution of disparate morphologies through deep time, including non-streamlined forms.

## Introduction

The fossil record documents how life evolved functional solutions to environmental challenges—from sudden catastrophes amid global mass extinctions, to pervasive obstacles that continue today. Through deep time, marine animals have faced these challenges while developing myriad solutions to navigating their physical environments (i.e., managing buoyancy, swimming efficiency, and maneuverability^1,2^). The most comprehensive macroscopic fossil record of functional anatomy from the past half-billion years belongs to animals that have nearly vanished from today’s seas: the externally shelled cephalopods (ectocochleates). Today, coleoid cephalopods (e.g., squid, octopus, and cuttlefish) are vital components of global ecosystems and human diet^3,4^. In contrast to their minor contributions today, ectocochleate cephalopods dominated trophic exchanges in many ancient ocean ecosystems. While coleoids are regarded as the most complex and mobile group of mollusks, the most fundamental swimming capabilities and selective advantages for the majority of cephalopods to ever exist (e.g., ammonoids and nautiloids) remain a mystery. The external conchs of these animals have long served as valuable index fossils across the globe^5,6^. However, these fossils may also provide key insights into the life habits and ecology of their once-living counterparts. Their external conchs constrain each internal component (the animal’s soft body and buoyancy chambers), allowing detailed reconstructions of various hydrostatic properties (buoyancy, life orientation, stability, and directional movement efficiency^7–11^). Moreover, the conch formed the animal-environment interface that dictated opportunity or defeat for a range of locomotion strategies^12–16^. By now, over a half-century of intensive paleo-ecological study has crystallized characterizations of ectocochleate swimming opportunities—or lack thereof—based on their external conch shapes^10,12,13,15–31^. A robust scheme linking morphology to ecological roles seems almost within reach, but an oversimplified approach will obscure how these animals have evolved solutions to the challenges imposed by environmental crises and day-to-day natural selection.

Accepted reconstructions of ancient cephalopod swimming capabilities are ripe for renewed investigation with emerging technologies. Decades of experiments and computations have illuminated some first-order relationships between conch morphology and hydrodynamics. First, and most intuitive, streamlined forms with compressed conchs and low whorl exposure (i.e., oxycones) incur lower hydrodynamic drag (specifically in turbulent flow regimes^12,13,18–20,28,32,33^). These morphologies are generally interpreted as nekton^24^, capable of reaching higher swimming speeds. In contrast, inflated forms (i.e., sphaerocones) incur higher hydrodynamic drag in turbulent flow, yet may be more efficient at smaller scales and/or velocities (i.e., lower Reynolds numbers^13,19,20,32^). Recent computer simulations demonstrate that serpenticones (forms exposing their earlier whorls) do not incur much more drag than oxycones, despite their complex flank topologies^20^. Therefore, conch inflation seems to impose a tradeoff on efficiency, which depends upon both size and speed. In addition to overall conch geometry, several second-order hydrodynamic factors complicate relationships between shape and hydrodynamics. Propulsive thrust relates to jet duration, frequency, and iteration^16,34^. Propulsive efficiency is tightly linked to the hydrostatics of posture and jet orientation^7,10,11,34–36^. Nuanced external shape features—keels; umbilical exposure; ornament of ribbing or spines^37^—produce substantial but nonlinear impacts on overall drag force^23^. Logical arguments and intensive computer simulations suggest that different combinations of first- and second-order conch shapes would cause radically different hydrodynamic opportunities for locomotion^12,13,15,17–20,23,28,32,33,38,39^. Many of the specific scenarios proposed, and trade-offs invoked, rely on reasoning that motivates renewed attention through rigorous quantification. Despite the immense body of work on ectocochleate cephalopod hydrodynamics, we remain uncertain whether the locomotion potentials of disparate conch morphologies were ever first-order controls on ecology, or an unavoidable target of natural selection.

**Figure 1:**
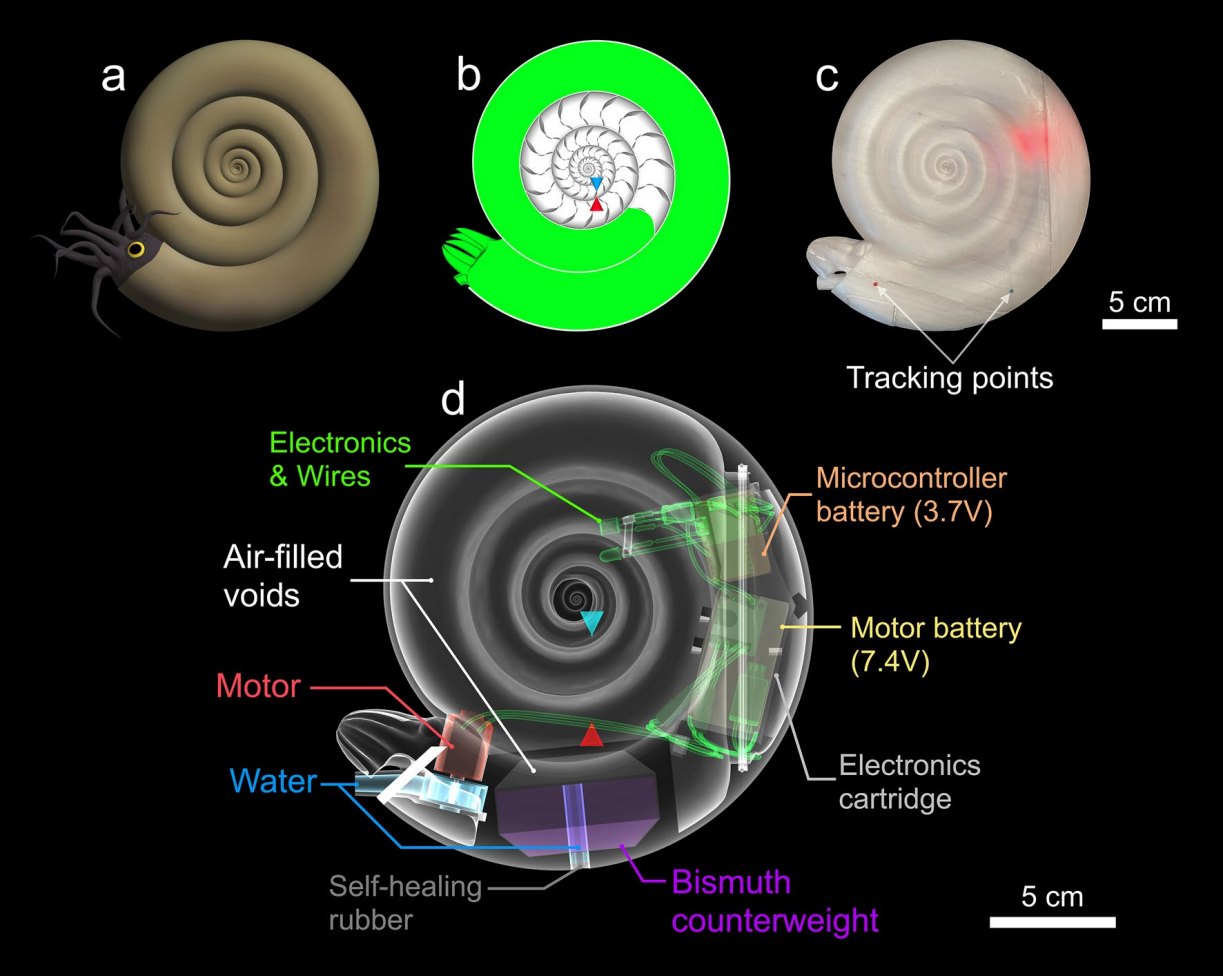
Biomimetic cephalopod robot design. (**a**) Hypothetical reconstruction of an ectocochleate cephalopod. (**b**) Virtual model used to determine the hydrostatic properties of this particular shape (serpenticone; see “Methods”). (**c**) Fully assembled 3D-printed robot with tracking points used in “3D motion tracking”. (**d**) Schematic of the assembled robot indicating each model component (color-coded by measured bulk density values; Table S1). The tips of the blue and red cones denote the locations of the centers of buoyancy and mass, respectively. All models were rendered in MeshLab^76^.

**Figure 2:**
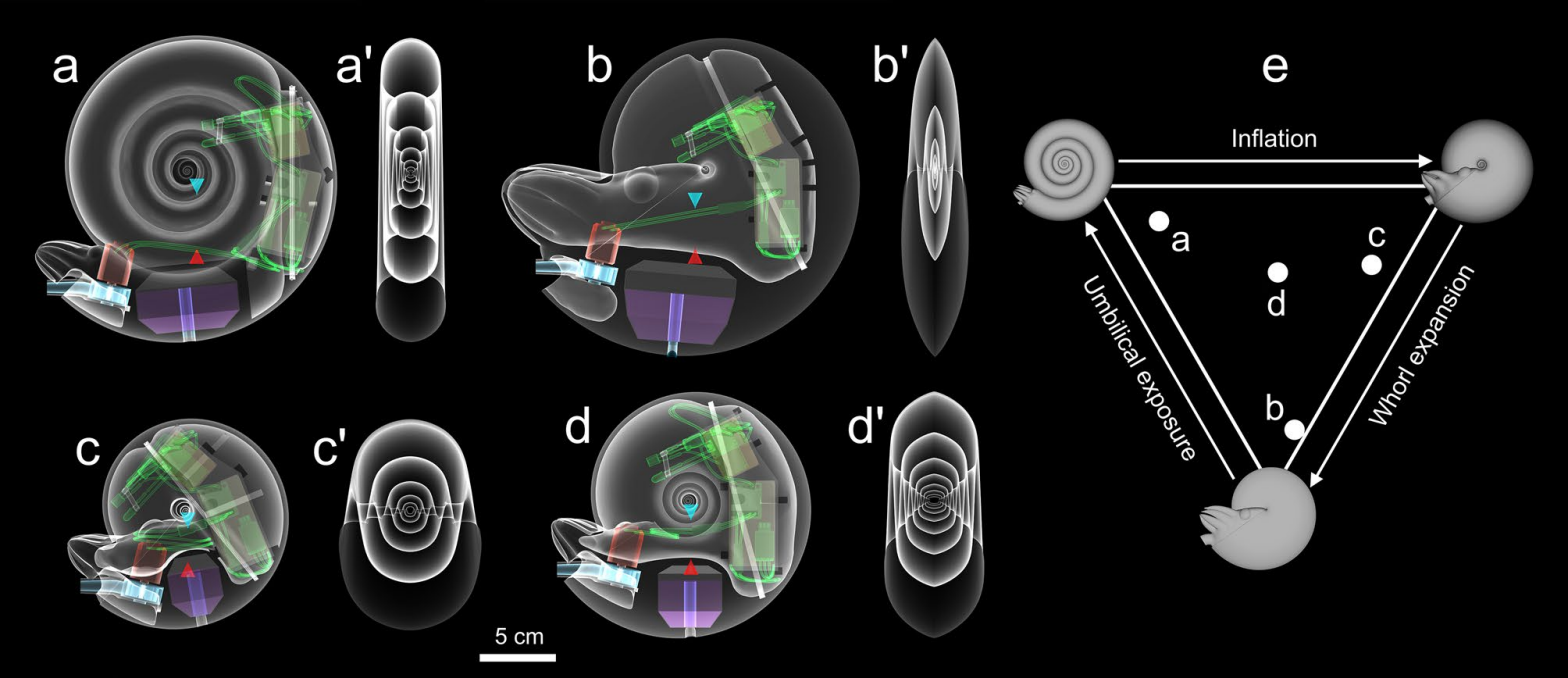
Transparent views of assembled biomimetic robots. (**a**) Serpenticone, (**b**) oxycone, (**c**) sphaerocone, and (**d**) morphospace center. Prime symbols (′) refer to transparent, transverse views of each robot’s external shape. All colors correspond to components on Fig. 1. The tips of the blue (upper) and red (lower) cones denote the centers of buoyancy and mass, respectively. Note that the separations of these hydrostatic centers are much larger than those reported in Table S3, creating artificially high stability to isolate the variable of conch shape and to minimize rocking. (**e**) Locations of each examined morphology on the Westermann morphospace^22^. The corners of this ternary diagram represent high whorl expansion (oxycone), high umbilical exposure (serpenticone), and high conch inflation (sphaerocone). All models were rendered in MeshLab^76^.

**Figure 3:**
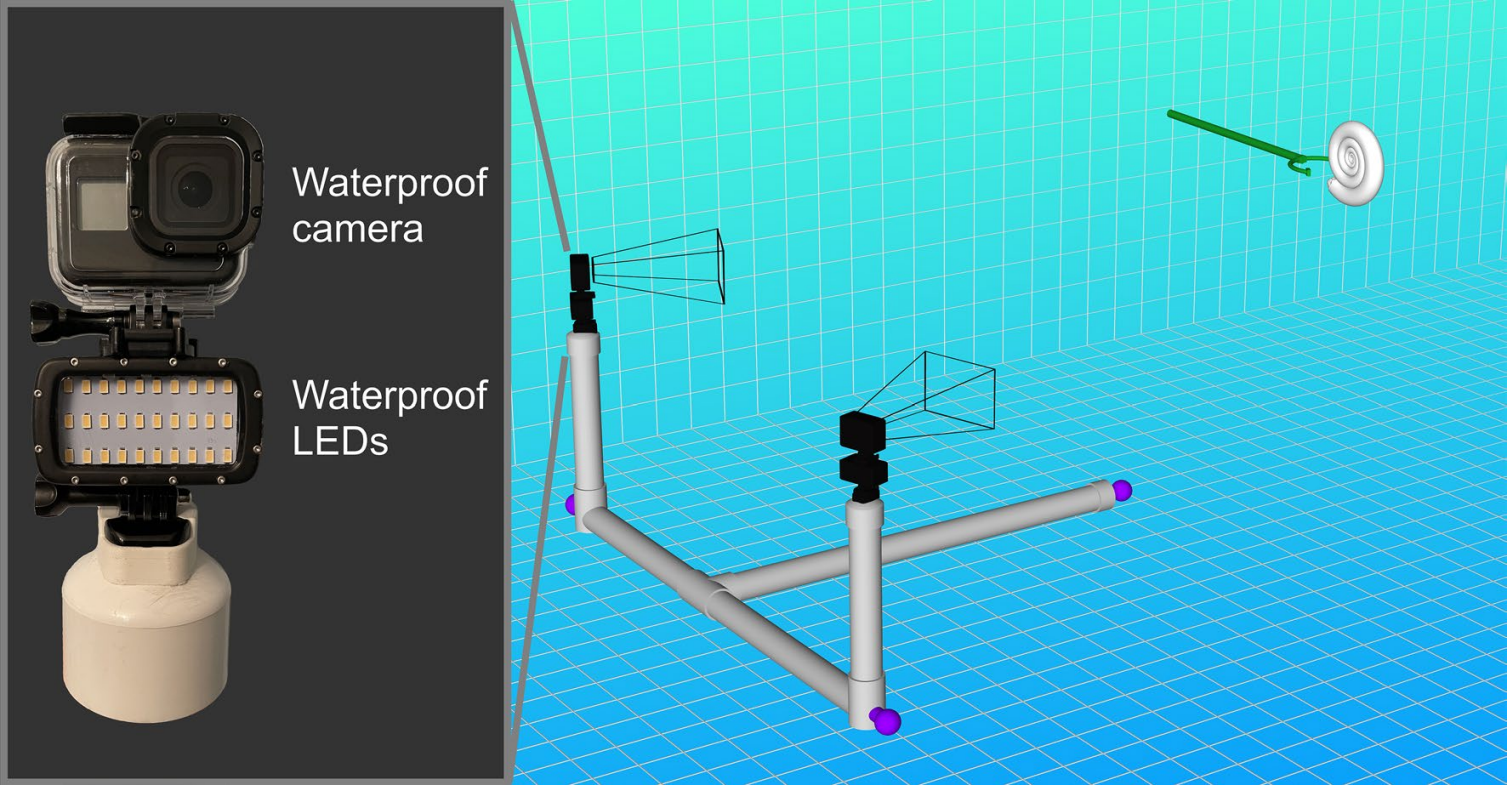
Schematic of the 3D motion tracking setup. A submersible camera rig consisting of a PVC skeleton (grey) and three steel counterweights (purple) allowed two waterproof cameras to be directed at each robot. A grabber tool (green) was used to position each robot, then send an infrared pulse through a fiber-optic cable to initiate jetting. The schematic (right) was rendered in MeshLab^76^.

We designed 3D-printed, biomimetic robots to test the practical locomotion consequences of ectocochleate conch shape (Fig. 1). Theoretical cephalopod conchs with disparate morphologies (Fig. 2) were constructed to investigate swimming capabilities across a broad range of an empirical planispiral morphospace^22^. Four models were constructed, consisting of three near-endmembers (serpenticone, Fig. 2a; oxycone, Fig. 2b; sphaerocone, Fig. 2c) and the morphospace center (Fig. 2d). These robots are near neutrally buoyant and assume their proper orientation in the water. Furthermore, they propel themselves with biologically relevant jet thrusts (similar to extant *Nautilus*^16^), allowing the comparative investigation of various kinematic properties. We removed the potential impacts of pitch reorientation by exaggerating each robot’s hydrostatic stability. Finally, we tested each robot in chaotic, real-world conditions. These approaches liberate the modeled cephalopods from being tethered in flow tanks or to force transducers, allowing more life-like investigations of syn vivo swimming capabilities with 3D motion tracking (Fig. 3). These approaches allow various physical properties to be monitored: acceleration from static initial conditions, coasting efficiency, jetting dynamics, hydrodynamic stability, and maneuverability.

We tested two hypotheses to evaluate the importance of conch shape for locomotion potential (at a particular scale). First, we investigate whether or not a compressed, involute morphology (oxycone; Fig. 2b) experiences significant reductions in drag compared to a compressed, evolute morphology (serpenticone; Fig. 2a), or an inflated, sphere-like form (sphaerocone; Fig. 2c). Second, we evaluate the hydrodynamic stability (coasting efficiency and course-stabilization) of each morphology, which should present a tradeoff^14,40^ between yaw maneuverability (ease or difficulty in turning about the vertical axis). The morphospace center may represent a generalist, experiencing physical properties intermediate to the near-endmembers. The null expectation would be that conch shape delivers unpredictable or insignificant differences in horizontal locomotion, coasting, and/or maneuverability (turning ability). Failure to reject this case would strengthen arguments that conch shape, while potentially influential on locomotion, would not be very useful to reconstruct the ecosystem roles of ectocochleate cephalopods or their evolutionary drivers through time. Alternatively, if these conch shapes produce substantial differences in swimming capabilities, they will elucidate the potential of using ectocochleate conch morphologies as proxies for life habits and tools to study evolutionary biomechanics.

## Results

Each of the biomimetic cephalopod robots have nearly identical volumes and masses (volumetrics and hydrostatics reported in Tables S1, S2, and S3), were nearly neutrally buoyant, and produce conservative *Nautilus*-like thrusts (Figs. S1 and S2; see “Thrust calibration” section of the methods). Additionally, these robots assume the proper orientation in the water, inferred from theoretical hydrostatic models (Table S4; see “Methods”). Designing the cephalopod robots (Fig. 2) with higher hydrostatic stability than their virtual counterparts effectively nullified the influence of hydrostatic stability on kinematics. That is, each model experienced low displacement angles from the vertical axis, generally ± 5° (Fig. S3). Under this condition, the models do not have to jet in alignment with their hydrostatic centers, and are not sensitive to the thrust angles inferred by the virtual hydrostatic models (Fig. 2; Tables S3 and S4). The robotic counterparts of the hydrostatically unstable morphotypes (serpenticone, sphaerocone, and morphospace center) are about an order of magnitude more stable, while the oxycone robot is around three times more stable (Table S4).

### Single-pulse, horizontal motility experiments

We choose to interpret drag consequences in terms of velocity and acceleration for each particular shape because they occupy nearly identical volumes and masses. Drag coefficients have been demonstrated to vary with conch size and/or swimming speed (Reynolds number) (see Fig. S5^13,18–20,41^) and do not appropriately quantify drag for these robots because they jet from a near stationary initial condition, reach some maximum velocity, then coast until they approach rest. A rough calculation of drag coefficients on the robots was performed by analyzing deceleration after ceasing jetting from a single pulse (see Supplementary Information text; Fig. S6; Table S5). The oxycone robot has the lowest computed drag coefficient of the four shapes (~ 0.14), while the sphaerocone has the highest coefficient (~ 0.61; Fig. S6E; Table S5). The serpenticone and morphospace center have similar computed drag coefficients of around 0.5 and cannot be statistically distinguished. Inflated morphotypes were more susceptible to yaw during horizontal movement, preventing analysis during later time steps where they approach rest. This tendency resulted in the modeled velocity curve (from which drag coefficients were determined; Fig. S6) to be likely overpredicted near the end of the captured motion (possibly reducing the computed drag coefficients). Other caveats with this approach include: rocking during movement (changes in pitch), low R-squared values between the recorded velocities and modeled curve for the oxycone and sphaerocone (Table S5), yaw during movement, and changes in added mass (acquiring mass by accelerating water in close proximity) at different velocities.

**Figure 4:**
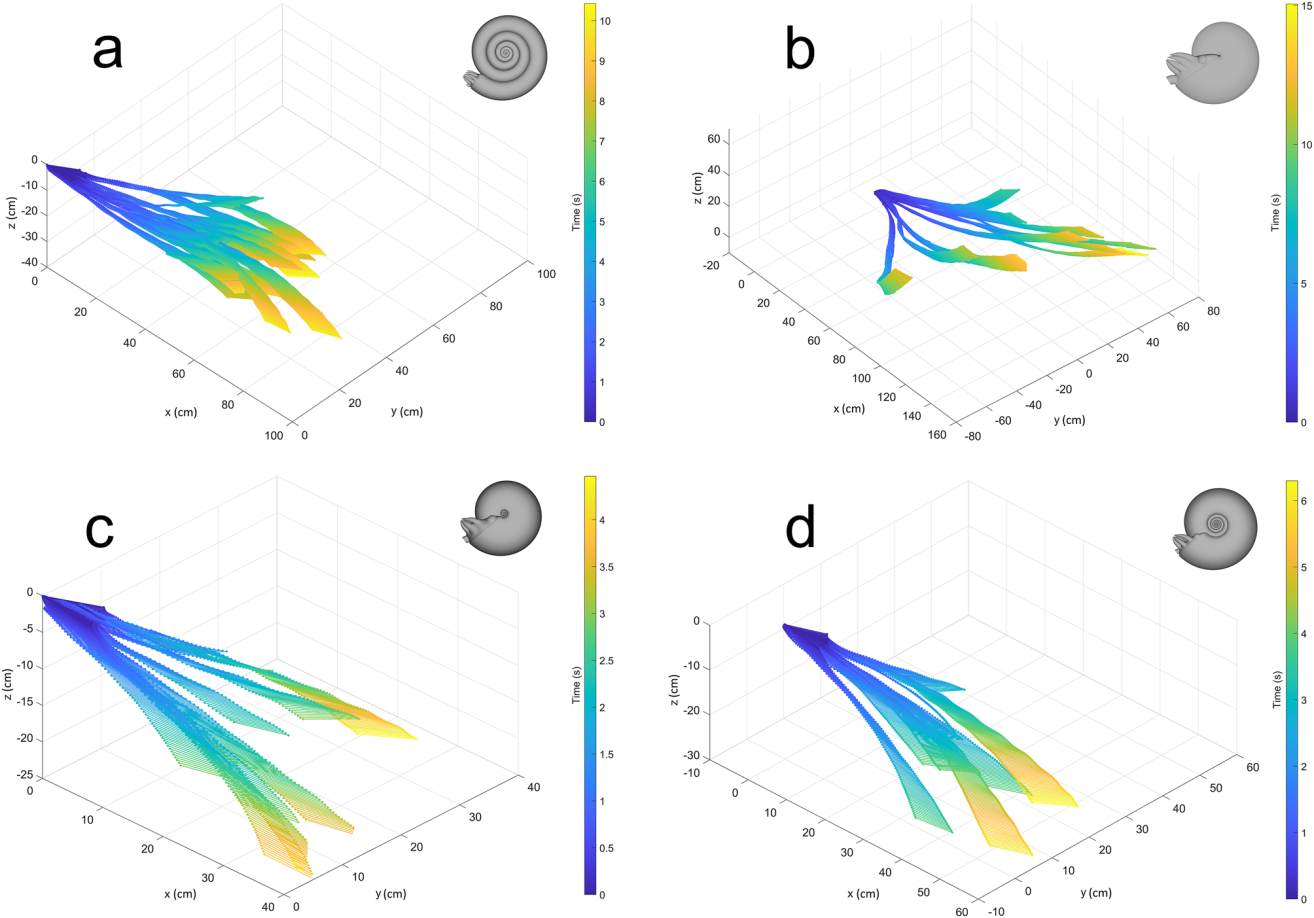
Three-dimensional positions of tracking points for each robot after initiating a single, one-second jet pulse. (**a**) Serpenticone, (**b**) oxycone, (**c**) sphaerocone, and (**d**) morphospace center. The times after initiating the motor are indicated by color.

**Figure 5:**
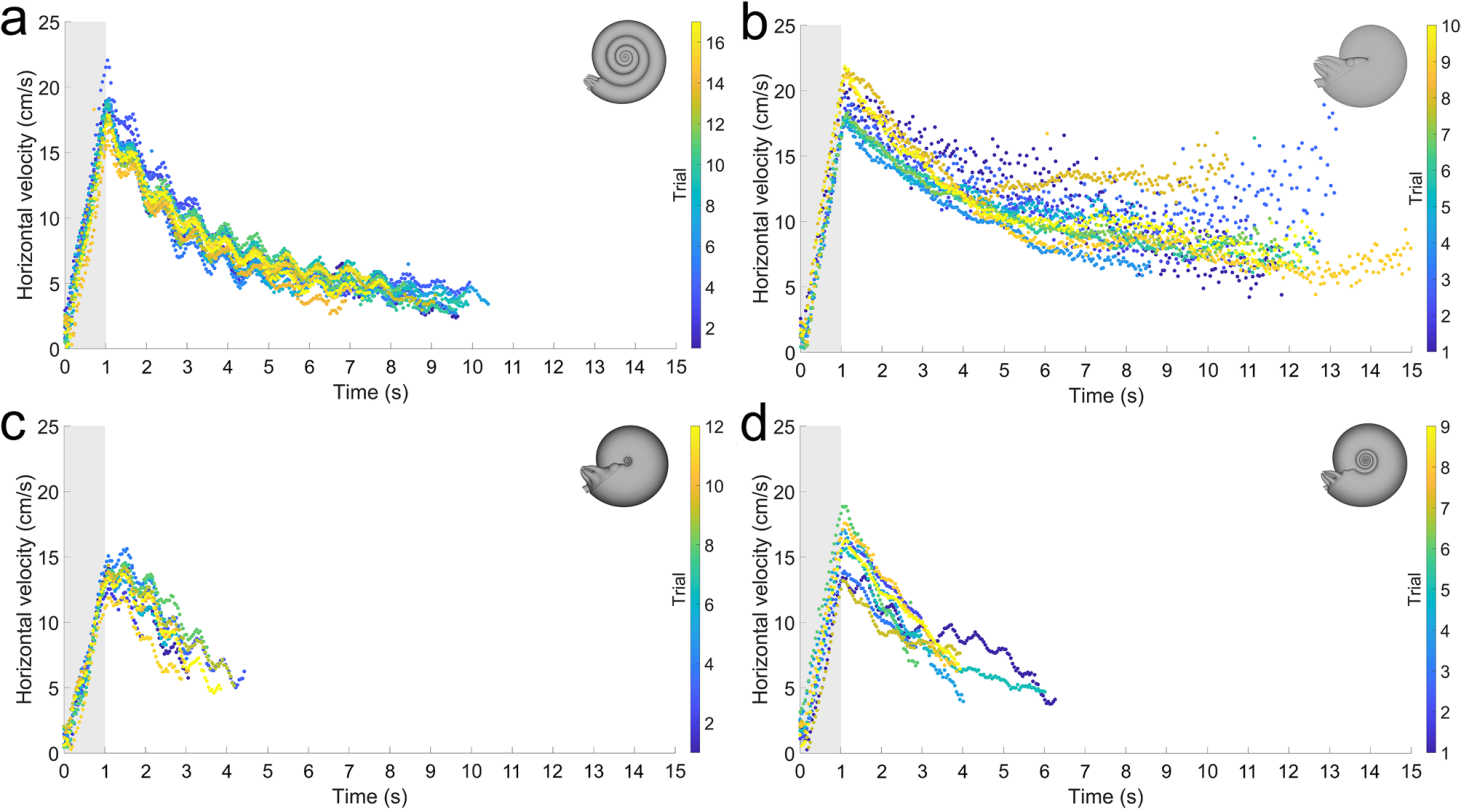
Velocities (horizontal components) for each model after initiating a single, one-second jet pulse. (**a**) Serpenticone, (**b**) oxycone, (**c**) sphaerocone, and (**d**) morphospace center. Shaded regions (first second) denote the time the motor was active. Colors indicate the trial performed for each robot.

After delivering a single, one-second jet pulse of equal thrust, backwards movement was recorded for each of the biomimetic robots (Fig. 4). Each morphotype displayed random lateral movement (perpendicular to the thrust direction) due to ambient currents in the experimental settings and slightly different initial conditions influencing yaw. Even with these variations in kinematics, velocity is well constrained for each of the examined morphotypes, and each trial follows strikingly similar patterns (Fig. 5). Even though rocking during movement is minor (Fig. S3), velocities oscillate during coasting. This behavior is largely due to the tracking points placed far from the model centers (Fig. S4), making recorded kinematics more sensitive to rocking behavior. Additionally, rotation away from the movement direction would cause increases in velocity during coasting, after which the models came to rest (e.g., the oxycone model; Fig. 5b).

Each of the examined morphotypes have distinct accelerations from a static initial condition (Table S6). While none of the 95% confidence intervals overlap for this metric, two main groupings can be derived. Compressed morphotypes (serpenticone and oxycone; 16.77 and 17.98 cm/s^2^, respectively) and inflated morphotypes (sphaerocone and morphospace center; 12.68 and 14.36 cm/s^2^, respectively). Within these groups, acceleration (accordingly, maximum velocity within this time window) is similar. However, compressed morphotypes consistently reach higher velocities (Table S7), demonstrating the consequence of conch inflation on hydrodynamic drag during acceleration from a static initial condition. The differences between the mean peak velocities of the serpenticone and oxycone are not statistically different according to a one-way ANOVA with a Games-Howell post hoc test (*p* > 0.05; Table S7). However, all other differences in mean peak velocities are significant below this level (Table S7).

### Three-pulse, horizontal motility experiments

**Figure 6:**
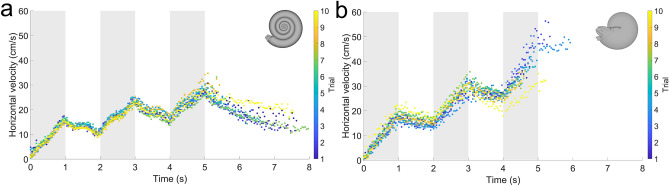
Velocities (horizontal components) for each model after initiating three, one-second jet pulses with one-second “refill” periods. (**a**) Serpenticone and (**b**) oxycone. Shaded regions denote the times the motor was active. Colors indicate the trial performed for each robot.

Inflated morphotypes experienced significant yaw after a single jet pulse, obscuring view of the tracking points at larger timesteps (Figs. 1 and S4). Consequently, experiments with three pulses (1 s duration and 1 s “refill” periods) were only performed on the compressed morphotypes (serpenticone and oxycone; Figs. 6, S8, S9; Table S8). After several pulses, velocities at the end of each cycle are further distinguished between the serpenticone and oxycone. The oxycone retains higher velocities while coasting during refill periods and reaches higher velocities. After multiple pulses hydrodynamic drag catches up to the serpenticone, preventing this morphotype from accelerating much higher velocities than the first pulse. Differences in acceleration between the one-pulse and three-pulse experiments (Tables S6 and S8) were likely due to performing each set of experiments in different settings (differences in ambient pool currents; subtle drifting before jetting).

### Hydrodynamic stability

The duration of each trial was governed by the view of the tracking points. Inflated morphotypes were recorded over shorter durations (Figs. 4 and 5) because they had the tendency to rotate about the vertical axis (yaw) obscuring tracking point views. Shortly after rotating away from the movement direction, these robots came to rest much more quickly than compressed morphotypes (Figure S10). The average trial durations between the sphaerocone and morphospace center cannot be statistically distinguished, however, all other combinations of morphotypes can be distinguished at the *p* < 0.001 level. The trial durations of the compressed morphotypes were at least twice as long as the inflated morphotypes. Compressed morphotypes not only reach higher peak velocities, but also coast longer and farther (Table S7). These properties are due to lower hydrodynamic drag and improved hydrodynamic stability during movement (preventing yaw).

### Yaw maneuverability

**Figure 7:**
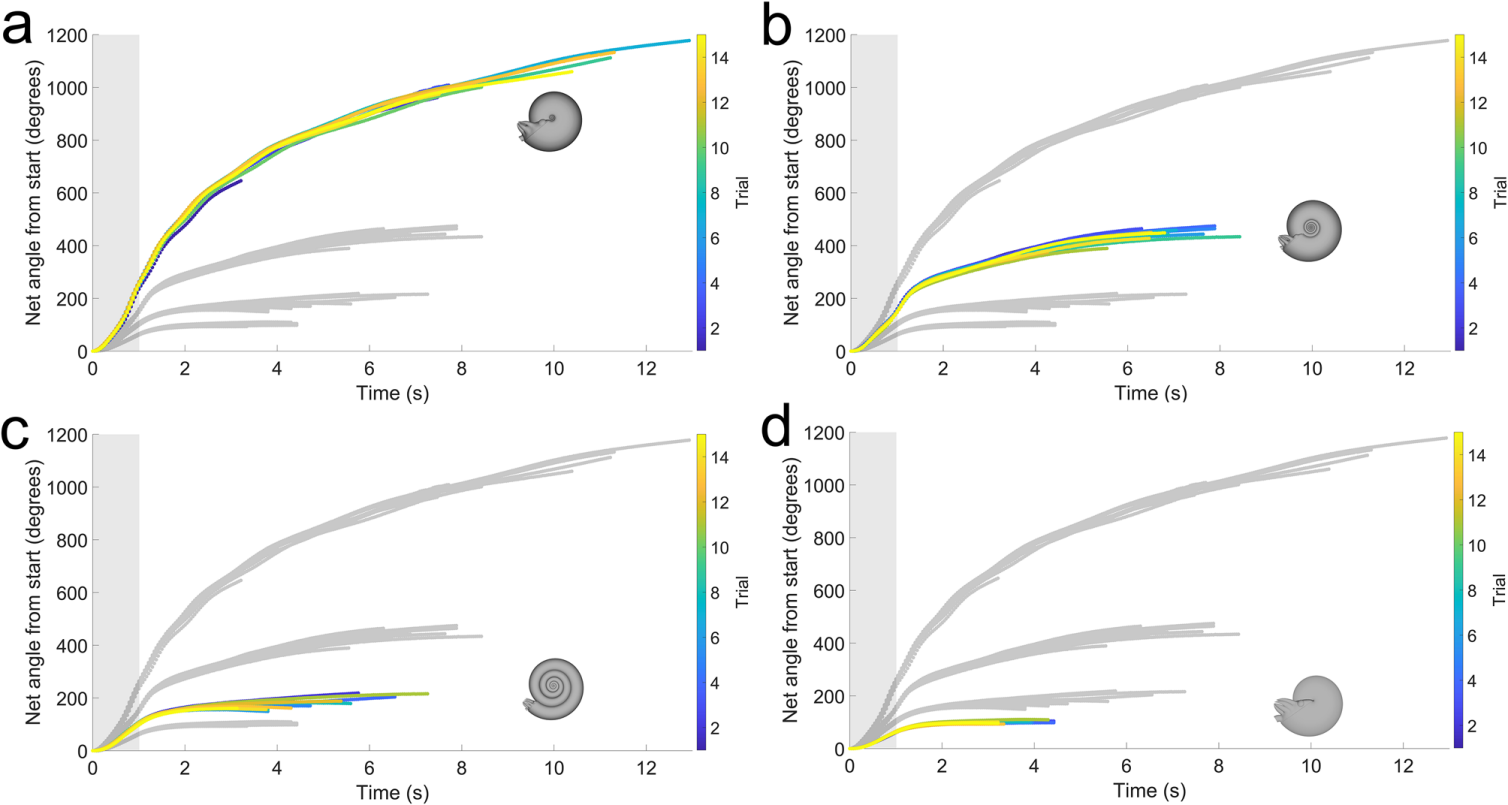
Maneuverability about the vertical axis (yaw). The net angle from starting position was recorded while monitoring rotation about the vertical axis. Each model experienced jet thrust oriented 90° to the lever arm passing between the hyponome and vertical axis passing through the hydrostatic centers. (**a**) Sphaerocone, (**b**) morphospace center, (**c**) serpenticone, and (**d**) oxycone. Shaded regions denote the time the motor was active (first second). Shaded curves denote the relative kinematics of each other robot. Colors indicate the trial performed for each robot (15 each).

**Figure 8:**
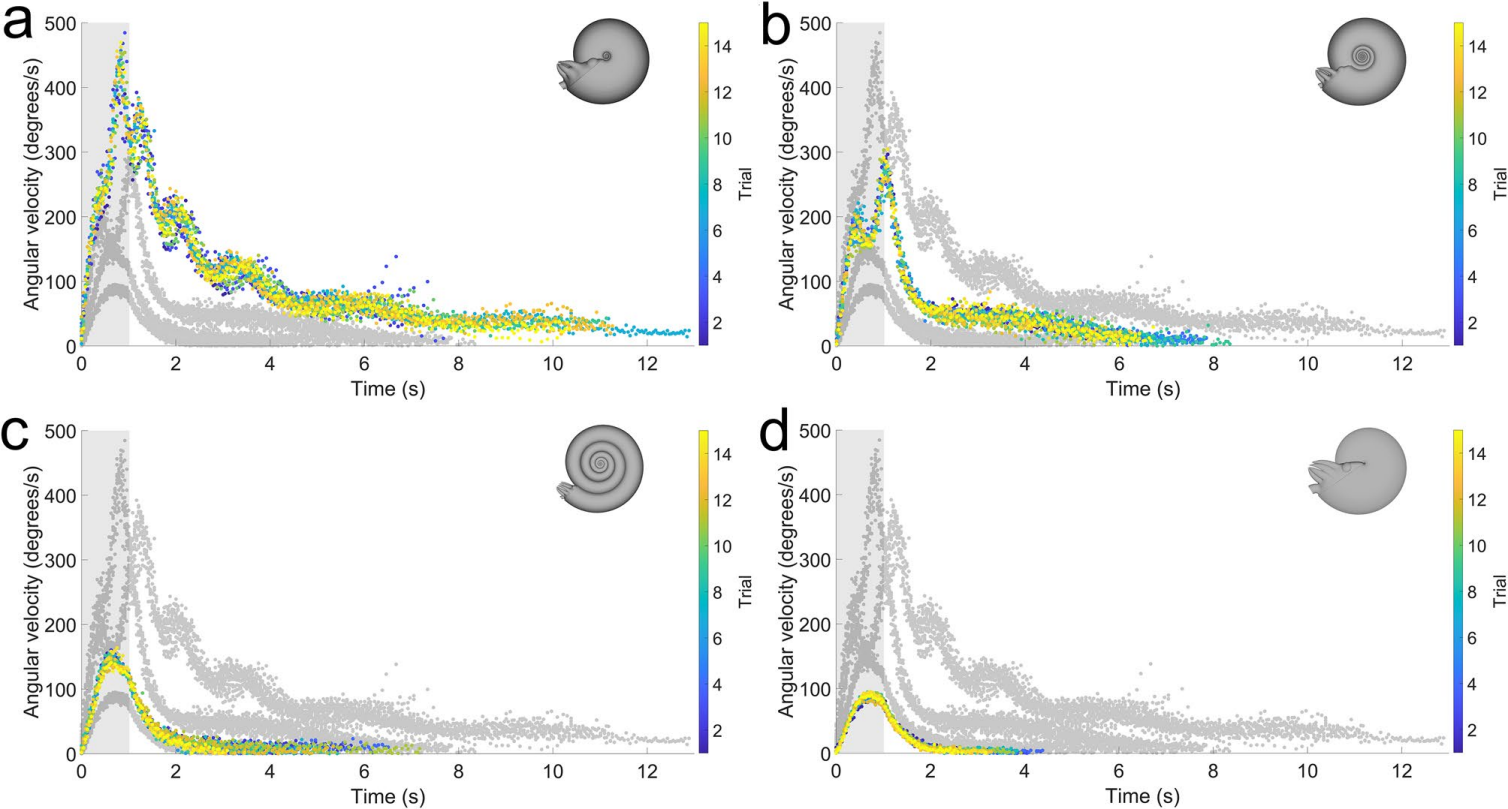
Angular velocity computed from rotation about the vertical axis (yaw). (**a**) Sphaerocone, (**b**) morphospace center, (**c**) serpenticone, and (**d**) oxycone. Shaded regions denote the time the motor was active (first second). Shaded curves denote the relative kinematics of each other robot. Colors indicate the trial performed for each robot (15 each).

After attaching a hyponome bent 90° and monitoring rotation about the vertical axis (yaw), each morphotype experiences consistent trials with distinct differences in net angle displaced (Fig. 7), and angular acceleration (Fig. 8). After a single pulse, each model rotates until asymptotically approaching some value (i.e., coming to rest). During a single, one-second pulse, inflated morphotypes (sphaerocone and morphospace center) rotate much further (> 3 revolutions for the sphaerocone) than compressed morphotypes (< 1 revolution). During jetting, the inflated morphotypes more quickly accelerate, and once jetting ceases, decelerate more slowly than inflated morphotypes (Fig. 8). The peak angular velocities (averaged over 15 trials) are statistically different between each morphotype according to an ANOVA (Table S9). Additionally, the sphaerocone reaches about five times the angular velocities of the oxycone during a one-second jet. The oscillations in angular velocity for the inflated morphotypes are likely due to the robots encountering their own wake from jetting and the turbulence generated from rotating the conch through the water.

Hydrodynamics (e.g., drag, wake dynamics, turbulence) dominate rotational resistance during the yaw experiments. Another influential property on rotational kinematics is the moment of inertia. Even though the hydrostatic centers between each robot and their virtual counterparts lie on the same vertical axes, the way in which their masses are distributed can influence their total moments of inertia. This property is most influential in a vacuum or media with low densities, or for shapes that incur low drag. Differences in this property between the robots and the virtual hydrostatic models (representing living animals with theoretical morphologies) were investigated by computing the moments of inertia for each component of unique density (see Supplementary Information text, Table S10). The moment of inertia is an additive property; therefore, the total moments of inertia are the sums of each of their components (Table S10). While the moments of inertia are considerably lower for the robot models (~ 20–30%), hydrodynamics still dominate rotational kinematics. The angular velocities, after one second of jetting, observed from the yaw experiments are about an order of magnitude lower than those computed in a vacuum (Table S11). When comparing the contribution of hydrodynamics for this calculation, using the moments of inertia for the robots and those representing the living animals, the differences in these proportions are minor (~ 2–12%). This approach demonstrates that the deviations from the proper moments of inertia in the robots do not considerably alter rotational dynamics during yaw movement.

The errors in tracking point locations for each set of experiments are reported in Table S12. Reproduced tracking point distances have standard deviations of less than 8.5 mm for each video recording.

## Discussion

While the current experiments represent only four datapoints across the planispiral morphospace and at a single size (~ 1 kg; Table S1), they illuminate some first-order, functional trends between disparate morphologies. Because these biomimetic robots are capable of freely moving in water, they allow the complex dynamics of movement to be investigated in response to jet thrust. Somewhat conservative, *Nautilus*-like jet thrusts (time-averaged value of ~ 0.3 N; Fig. S2) yield biologically relevant scenarios for comparing the hydrodynamic consequences of different conch shapes. When jetting from static initial conditions, compressed morphotypes (serpenticones and oxycones) reach higher velocities than inflated morphotypes. At the examined scale, the serpenticone robot reaches velocities similar to the oxycone despite its more complex flank topology (Fig. 5). Only after multiple pulses, does the extra drag produced by this less streamlined shape create substantial differences (Fig. 6). This behavior corroborates the results of computational fluid dynamics simulations in virtual settings^20^, and does not support binning serpenticones as planktic organisms^24^. The kinematic differences between the robots may reflect differences in metabolic constraints for their living counterparts, with compressed morphotypes expending less energy to move through the surrounding water. However, the living animals represented by each of these morphotypes were likely much slower than fish or coleoids of equivalent size (~ 10–25% for *Nautilus*^16^). Therefore, swimming speed is probably not the best metric of performance for ammonoids, nautiloids, and other ectocochleates. Some shapes (e.g., orthocones) may have been able to experience relatively high velocities during escape jetting, but only in one direction^36^. Recent studies demonstrate that *Nautilus* experiences low metabolic cost of locomotion at low velocities despite the “inefficiency” of jet propulsion compared to undulatory swimming^34^. This perspective for extinct ectocochleates might be beneficial because different conch shapes would have imposed different metabolic costs for particular modes of locomotion. Though, this relationship is complicated due to the vast taxonomic diversity of ectocochleate cephalopods, likely reflecting similar degrees of physiological differences between clades as well as morphological disparities of the soft body^42,43^.

Some of the most striking hydrodynamic differences between each of the robots are their variable coasting distances (Fig. 5; Table S7). Compressed morphotypes are able to coast much further on a single jet compared to inflated morphotypes. This capability is controlled by directional streamlining (drag) and also hydrodynamic stability. The latter property is the result of compressed objects resisting turning through the water (yaw in this case). When experiencing yaw, these shapes would experience high pressures along their flanks, whereas sphere-like shapes would more easily spin^14^. These results suggest that inflated cephalopods like sphaerocones would have to jet more periodically to make corrections in trajectory during movement. The hydrodynamic stability of compressed morphotypes also reflects superior steerage^14,24^ (i.e., travelling opposite of the thrust vector). This ability was also likely improved for taxa with distinct keels (e.g., many Pinacoceratidae, Prolecanitida Oppeliidae, Sphenodiscidae, among others). These more nuanced properties could be illuminated through particle image velocimetry (PIV) in future studies.

Coasting ability during horizontal movement is inversely related to yaw maneuverability. By orienting thrust 90° to the lever arm passing between the robot hyponome and hydrostatic centers, inflated morphotypes more easily rotate in response to the same jet thrust (Figs. 7 and 8). Because all models have nearly equal volumes and masses, compressed morphotypes have larger conch diameters (Table S7). These larger diameters create larger lever arms, which would give these shapes rotational advantage (higher torque) if they were in a vacuum. However, in water, the broad flanks of compressed morphotypes create substantial rotational drag compared to sphere-like morphotypes, attenuating rotation and acceleration. These differences highlight a physical tradeoff between hydrodynamic stability and yaw maneuverability, presenting several advantages to cephalopods with sphere-like conchs. These shapes would have been able to rotate about the vertical axis much more quickly, denying soft body access to small predators. Furthermore, these shapes would allow 360° access to prey items closely surrounding these cephalopods while simultaneously minimizing self-generated wake and energy expenditure. Some heteromorph ammonoids may have had similar turning capabilities about the vertical axis, but with higher hydrostatic stability compared to sphaerocones^31^.

Hydrodynamic stability improves the regulation of trajectory and the resistance to external forms of energy (e.g., wake generated by other animals, wave energy, and flow at the interfaces of bathymetric features). Similarly, hydrostatic stability limits rocking about the horizontal axes but makes it more difficult to modify orientation. Fish generally have much lower hydrostatic stability compared to ectocochleate cephalopods^17^, but manage stability and maneuverability with the dynamic beating of fins^44,45^ and with different body shapes^40,44,46^. In contrast, ectocochleate cephalopods are mostly rigid bodies propelled by jet thrust, with some hydrodynamic contributions made by soft body orientations^39^. Therefore, conch shape primarily governs how ectocochleates interact with the physics of their external environments. Perhaps this constraint also influenced the habitat occupation of different morphotypes. Streamlined, compressed cephalopods have been documented in shallower paleoenvironments, with less stable, evolute and/or inflated morphotypes^7,14,24,47^ reported from more distal settings^12,32,48,49^. These patterns are also well documented in terms of intraspecific variation (i.e., Buckman’s Rules of Covariation)^50,51^. These cases may reflect ecophenotypic responses to high or low energy habitats or constraints on life habits in these different settings. However, opposite patterns^52^ and more ambiguous patterns^47^ have been reported, which complicate this trend. In addition to first-order conch shape, the hydrodynamic influences of conch ornamentation (e.g., coarse ribs) may be responsible for obscuring some these trends, and should be considered in future research.

The abundance of non-oxyconic shapes in the fossil record suggests that streamlined, involute conchs do not represent a single optimum morphology. Departures from this shape are expected because there is no morphology that is universally adapted to being the “best” swimmer^2,13,40,44^. To better understand ectocochleate cephalopod life habits and functional morphology, we should focus on evaluating the different performances of these shapes, not solely limited to hydrodynamic drag incurred while swimming backwards. That is, a synthesis of hydrostatics, hydrodynamics, and the related physical tradeoffs of these properties can improve the use of these animals as tools to study evolutionary biomechanics. Through the fossil record, ectocochleates have preserved a massive dataset of conch shapes, and how they have changed over most of the current eon. These cephalopods have substantially shifted their occupation of their morphospace through time, especially during mass extinctions^37,53–57^. A more complete understanding of their life habits and physical constraints would provide important context to the ecological roles of these animals and their evolutionary biomechanics in response to environmental perturbations throughout the Phanerozoic.

Differences in hydrodynamic stability and yaw maneuverability highlight some of the hydrodynamic constraints imposed on life habit by disparate conch shapes. Stability-maneuverability tradeoffs are faced by many organisms^40,44–46,58^, and should receive more attention with regard to ectocochleate functional morphology. High hydrodynamic stability^14,20^ of oxycones suggests that ammonoids approaching this endmember had higher motility and coasting efficiency, inferring more active lifestyles. The higher hydrostatic stability of oxycones (and extant nautilids) improves their directional efficiency of locomotion. These morphotypes are less sensitive to jetting in alignment with their hydrostatic centers because they have stronger restoring moments confining them to some preferred orientation. In addition to functional constraints, higher activity life habits for these morphotypes are supported by biotic^25^ and lithofacies^47,48^ associations as well as isotopic analyses^25,59^. The horizontal motility experiments in the current study demonstrate that serpenticones and sphaerocones are not necessarily restricted to planktic life habits^24^. While these morphotypes would have had much lower hydrostatic stability than extant *Nautilus* (~ 35% and 14%, respectively; Table S4), they still would have been able to move at comparable velocities to the biomimetic robots if they could produce *Nautilus*-like jet thrust. However, these less stable morphotypes would be more sensitive to jetting at the angle where the thrust vector passes through the hydrostatic centers. Thrust angles for these morphotypes would most efficiently transmit jet thrust at diagonally upward angles (~ 43° for serpenticones and ~ 33° for sphaerocones with idealized conch parameters; Table S4). Nevertheless, the current experiments infer advantages in locomotion for morphotypes commonly regarded as hydrodynamically inferior. Furthermore, the many cephalopods that display intermediate conch geometries may represent generalists that do not accel in any particular swimming capability, but have intermediate performances for both stability and maneuverability.

The sensitivity to these thrust angles can be experimented upon in future studies, but would require engineering of models with submillimeter-level accuracy in the placement of the total center of mass (Table S3). Such experiments would add important context to the directional efficiency of locomotion for disparate conch shapes. Vertical migration in the water column is a fundamental habit for many extant cephalopods^60^, including the nautilids^61^. Rather than changing buoyancy for these behaviors, extant nautilids rely on active locomotion^61^. While extinct ectocochleates had disparate internal characteristics that could have influenced the function of their hydrostatic apparatus^62,63^ (e.g., septal and siphuncular morphologies), it is parsimonious to suggest that extinct ectocochleates (including ammonoids) relied on active swimming as well for vertical movements. An investigation of the relationships between hydrostatic and hydrodynamic properties is necessary to fully understand the directional swimming capabilities of particular morphologies.

The current study serves as a baseline for ectocochleate cephalopod swimming capabilities because only first-order conch shape (coiling) is considered. Planispiral cephalopods (especially ammonoids) experimented with myriad second-order features^37^, including various ornamentation patterns (ribs, keels, spines, nodes, tubercles, etc.), as well modifications of the aperture (e.g., varices, constrictions, lappets, and rostra). Each of these shapes would have had hydrodynamic consequences^13,23,33^, modifying the physical properties of the fundamental conch shape. Furthermore, the hydrodynamic properties of particular shapes, in terms of directional swimming and maneuvers, are dependent upon size^13,19^. Ectocochleates had to navigate changing physical properties throughout ontogeny, while responding to various physical tradeoffs. Finally, as we learn more about the soft bodies of these animals^43,64,65^, the functional advantages and consequences of potentially disparate morphologies can be further explored (e.g., differences in external shape, propulsive efficiencies, and musculature).

Reexamining the ectocochleate cephalopod morphospace^22^ in the context of functional tradeoffs will prove useful for interpreting the life habits, selective advantages, and physical constraints of animals that were key components of marine ecosystems for hundreds of millions of years. While planispiral cephalopods display a narrower range of physical properties compared to their uncoiled ancestors^9,26^, or heteromorph ammonoids^10,11,35,36,66^, their conchs served as interfaces between their physical environments and imposed different physical constraints depending on their shapes. Consequently, these conch morphologies represent functional solutions to the various challenges of navigating these environments—likely influencing the life habits of individual animals, their ecological roles, and selective pressures through deep time.

## Methods

### Virtual hydrostatic model parameters

**Figure 9:**
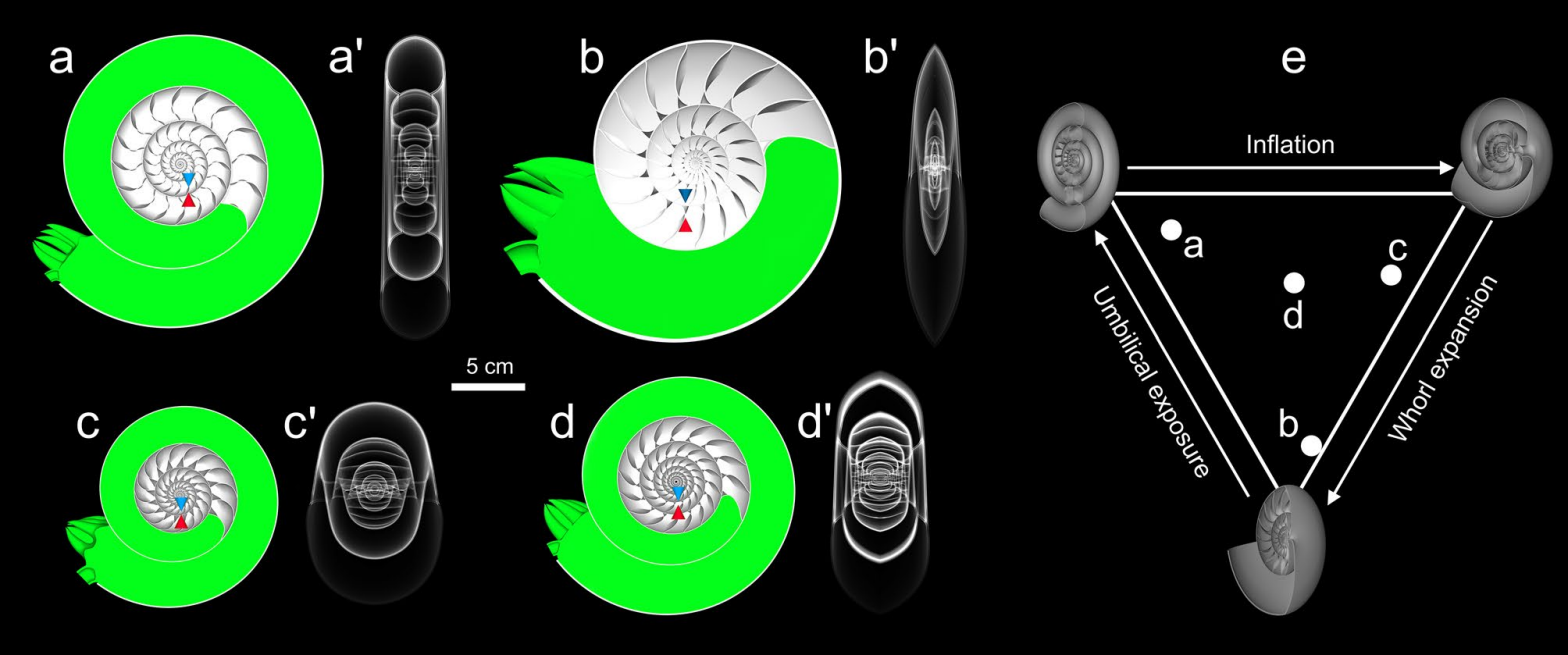
Hydrostatic models of theoretical planispiral cephalopods. These models were constructed by morphing a *Nautilus pompilius* conch into ammonoid shapes (see “Methods”): (**a**) oxycone, (**b**) serpenticone, (**c**) sphaerocone, and (**d**) morphospace center. The centers of buoyancy and mass are denoted by the tips of the blue (upper) and red (lower) cones. Prime symbols (′) refer to transparent, transverse views of each respective conch shape. (**e**) Westermann morphospace^22^ showing relative positions of these conch shapes. All models were rendered in MeshLab^76^.

Various morphological characteristics were held constant in order to isolate and manipulate the variable of conch shape. A CT-scanned *Nautilus pompilius* conch was essentially morphed into ammonoid-like conch shapes, populating the Westermann morphospace^22^ while holding constant septal morphology, septal spacing, and shell/septal thicknesses (Fig. 9). Furthermore, body chamber proportions were determined by iteratively computing soft body volumes that yield *Nautilus*-like chamber liquid (~ 12% of the phragmocone volume retained)^67,68^. Septal spacing was measured as the angle from the ventral attachment of the current and previous septa, and the spiraling axis of the conch. Because septal spacing differs in early ontogeny (Fig. S11), only measurements from the 7th to 33rd (terminal) septum were considered. The average angle of 23.46° ± 3.32° (standard deviation) was rounded to 23° and held constant throughout the ontogeny of the hydrostatic models.

Shell and septal thicknesses were measured with digital calipers from a physical specimen of *Nautilus pompilius* (Table S13). These measurements were recorded as a ratio of inner whorl height (measured from the ventral point on the current whorl to the ventral point on the previous whorl). These ratios were used in the theoretical models to define shell and septum thicknesses (3.1% of inner whorl height for shell thickness and 2.1% of inner whorl height for septal thickness; Table S13).

### Hydrostatic model construction

The near-endmember models were constructed from representative ammonoid specimens (*Sphenodiscus lobatus* and *S. lenticularis*—oxycone; *Dactylioceras commune*—serpenticone; *Goniatites crenistria*—sphaerocone). Lateral and transverse views were measured from figured specimens for the oxycone (Fig. 5 of Kennedy et al.^69^), serpenticone (Fig. 2 of Kutygin and Knyazev^70^), and sphaerocone (Figs. 17 and 20 of Korn and Ebbighausen^71^). These models were constructed with array algorithms similar to earlier hydrostatic models^9,35,72^, which were used in a piecewise manner to account for allometric changes in coiling throughout ontogeny (Table S14). These arrays replicated the adult whorl section backwards and translated, rotated, and scaled each successive one. These whorl sections were bridged together to create a single tessellated surface representing the outer interface of the shell. Shell thickness was defined by shrinking the original whorl section so that the thickness between the two was equal to 3.1% of the inner whorl height (Table S13), then using the same array to build the internal interface of the shell. The morphospace center was constructed from previously used conch measurements^18^ and averaging the whorl section shape in blender (Fig. S12). The corresponding Westermann morphospace parameters (Fig. S13) for each morphology are reported in Table S15.

Virtual models of the septa were derived from the CT-scan of *Nautilus pompilius* (Fig. S14). A single septum was isolated from the adult portion of the phragmocone then smoothed to delete the siphuncular foramen. This septum was placed within the whorl section of each theoretical model and stretched in the lateral directions until it approximately fit. The “magnetize” tool in Meshmixer (Autodesk Inc.) was used to attach the septal margin to the new whorl section so that the *Nautilus* suture was transferred to the new whorl section. The septum was then smoothed to reconcile the first order curves with the new location of the septal margin. The respective septum for each theoretical model was then replicated with the same array instructions used to build the shell. Because each replicated object was rotated one degree (Table S14), 22 septa were deleted in between every two so that the septal spacing was equal to 23° (Fig. S11).

For each theoretical model, the septa were unified with the model of the shell using Boolean operations in Netfabb (Autodesk Inc.). To perform hydrostatic calculations, virtual models must be created for each material of unique density. The virtual model of the shell constrains the shape of the soft body (within the body chamber) and chamber volumes (within the phragmocone). These internal interfaces were isolated from the model of the shell, then their faces inverted for proper, outward-facing orientations of their normals. A conservative soft body estimate was created, aligning with previously published reconstructions^64,65,73^. The profile shape of this soft body was scaled and maintained between each model. External interfaces of the shell and soft body were also isolated to create a model of the water displaced by each theoretical cephalopod. Each of these models are necessary for hydrostatic calculations (buoyancy and the distribution of organismal mass).

Each hydrostatic model is stored in an online repository (Dataset S1; https://doi.org/10.5281/zenodo.5684906). The hydrostatic centers of each virtual model and their volumes and masses are listed in Tables S16 and S17.

### Hydrostatic calculations

Each theoretical model was scaled to have equal volume (near one kilogram; 0.982 kg–a result of arbitrarily scaling the sphaerocone model to 15 cm in conch diameter). An object is neutrally buoyant when the sum of organismal mass is equal to the mass of water displaced (the principle of Archimedes). The percentage of chamber liquid can be computed to satisfy this condition.

1 $${\Phi } = \frac{{\left( {\frac{{{\text{V}}_{{{\text{wd}}}} {\uprho }_{{{\text{wd}}}} - {\text{V}}_{{{\text{sb}}}} {\uprho }_{{{\text{sb}}}} - {\text{V}}_{{{\text{sh}}}} {\uprho }_{{{\text{sh}}}} }}{{{\text{V}}_{{{\text{ct}}}} }}} \right) - \left( {{\uprho }_{{{\text{cl}}}} } \right)}}{{\left( {{\uprho }_{{{\text{cg}}}} - {\uprho }_{{{\text{cl}}}} } \right)}}$$

where V_wd_ and ρ_wd_ are the volume and density of the water displaced, V_sb_ and ρ_sb_ are the volume and density of the soft body, V_sh_ and ρ_sh_ are the volume and density of the shell, ρ_cl_ is the density of cameral liquid, ρ_cg_ is the density of cameral gas, and V_ct_ is the total volume of all chambers. A soft body density of 1.049 g/cm^3^ is used based on bulk density calculations of *Nautilus*-like tissues^74^, a seawater-filled mantle cavity, and thin calcitic mouthparts^21^. A shell density of 2.54 g/cm^3^^74^, cameral liquid density of 1.025 g/cm^3^^75^, and cameral gas density of 0.001 g/cm^3^ are adopted from recent hydrostatic studies.

Other hydrostatic properties depend on the relative positions of the centers of buoyancy and mass. The center of buoyancy is equal to the center of volume of water displaced. This center and the centers of each virtual model of unique density were computed in the program MeshLab^76^. The individual centers for each organismal model (soft body, shell, cameral liquid and cameral gas) were used to compute the total center of mass, with an average weighted by material density:

2 $$M = \frac{{\sum \left( {L*m_{o} } \right)}}{{\sum m_{o} }}$$

where M is the total center of mass in a principal direction, L is the center of mass of a single object measured with respect to an arbitrary datum in each principal direction, and $m_{o}$ is the mass of each object with unique density. Equation 2 was used in the x, y, and z directions to compute the 3D coordinate position of the center of mass. The centers of mass for the chamber contents (liquid and gas) were set equal to the center of volume of all chambers, a minor assumption considering the capillary retention of liquid around the septal margins in the living animals^62^.

The hydrostatic stability index (S_t_) is computed from the relative location of the centers of buoyancy (B) and mass (M), normalized by the cube root of volume (V) for a dimensionless metric that is independent of scale:

3 $$S_{t} = \frac{{ \sqrt {\left( {B_{x} - M_{x} } \right)^{2} + \left( {B_{y} - M_{y} } \right)^{2} + \left( {B_{z} - M_{z} } \right)^{2} } }}{{\sqrt[3]{V}}}$$

where the subscripts correspond to the x, y, and z components of each hydrostatic center.

Apertural orientations were measured in blender after orienting each model so that the center of buoyancy was vertically aligned above the center of mass. Apertural angles of 0° correspond to a horizontally facing soft body, while angles of + 90° and − 90° correspond to upward- and downward-facing orientations, respectively.

Thrust angles were measured from the hyponome location (ventral edge of the aperture) to the midpoint of the hydrostatic centers, with respect to the horizontal. Thrust angles of 0° infer idealized horizontal backward transmission of energy into movement, while thrust angles of + 90° and − 90° infer more efficient transmission of energy into downward and upward vertical movement, respectively.

### Biomimetic robot construction

To isolate the variable of shell shape on swimming capabilities, only the external shape, and static orientation of each virtual hydrostatic model were used to build physical, 3D printed robots. That is, each model has artificially high hydrostatic stability (Tables S3) to nullify the effect of the thrust angle (the angle at which thrust energy passes through the hydrostatic centers and most efficiently transmits energy into movement; Table S4). Less stable morphotypes (e.g., serpenticones and sphaerocones) are more sensitive to the constraints imposed by this hydrostatic property.

**Figure 10:**
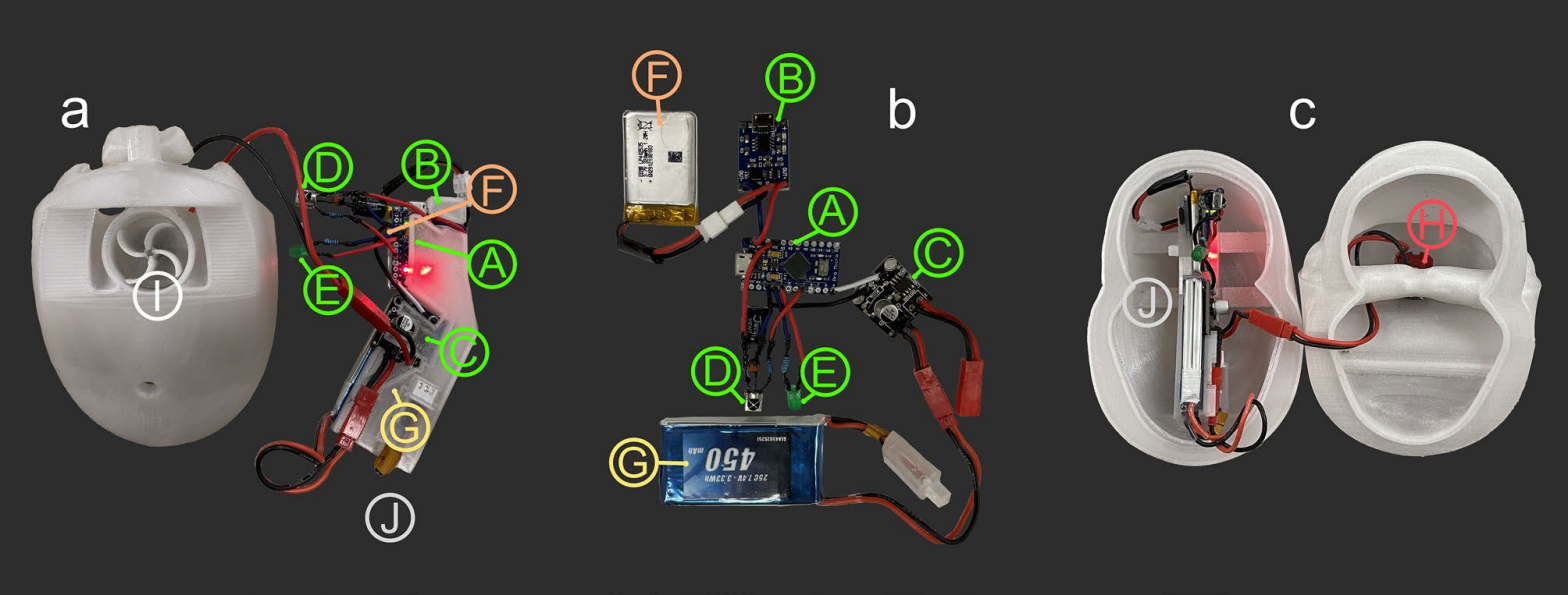
Biomimetic cephalopod robot components. (**a**) Ventral view of the sphaerocone biomimetic robot (before covering the pump and mantle cavities) with assembled electronics cartridge to the right. (**b**) View of electronic components that fit into the cartridge. (**c**) Electronics cartridge placed in robot. These two halves are fit together with wax to create a water-tight seal. Each model component is denoted by letters in circles: A = Arduino microcontroller, B = microcontroller charger / voltage regulator, C = motor driver, D = infrared sensor, E = indicator LED, F = microcontroller battery (3.7 V), G = motor battery (7.4 V), H = brushed motor, I = impeller and water pump cavity, J = electronics cartridge. The colors of annotations correspond to components depicted in Figs. 1 and 2.

Space constraints inside each model were determined by first constructing a propulsion system and electronic components that operate the motor. The models use impeller-based water pumps (Figs. 1d and 10a) driven by a brushed DC motor. This system creates a partial vacuum by centrifugal acceleration, drawing water from a “mantle cavity” and expelling it out of a “hyponome”. This system was optimized by iteratively designing models in Blender^77^, then testing 3D-printed, stand-alone water pumps. After three iterations, a four-blade impeller and gently tapering hyponome (inner diameter at distal end = 6.7 mm) were chosen. The electronic components used to drive the motor consist of an Arduino Pro Micro microcontroller, a motor driver, and two batteries (Fig. 10). A 3.7 V battery operates the microcontroller, and a larger 7.4 V battery supplies power to the motor. Communication is achieved via infrared, allowing specification of the jet pulse duration, number of pulses, and the power level of the motor (using pulse-width modulation; PWM). Each of these electronic components fold into a compact cartridge capable of being plugged into 3D-printed models of each investigated shell shape (Figs. 2 and 10). Each model was designed with brackets to hold the electronics cartridge in place. The sphaerocone had the most severe space constraints, with low conch diameter to volume ratio. After determining the space required for the electronics (Fig. 10) this model was scaled to 15 cm, and all other models were scaled to have similar volumes (with subtle volume differences due to minor differences in soft body shape compared to the hydrostatic models).

In addition to having a propulsion system, biomimetic cephalopod robots must also be capable of neutral buoyancy, while assuming the proper orientation in the water. These robots, and their once-living counterparts, each have differing material densities and associated mass distributions for each component. To reconcile these differences, the total mass and total centers of mass for each model were manipulated by controlling the volume and 3D distribution of the 3D-printed PETG (polyethylene terephthalate glycol) thermoplastic. That is, the shape of this material holds each model component in place while correcting for these differences in hydrostatics. The PETG mass required for neutral buoyancy was found by subtracting the mass of every other model component from the mass of the water displaced by the model (i.e., electronics cartridge, bismuth counterweight, liquid, motor, batteries, electronic components, and self-healing rubber; Table S1). This model configuration also allows buoyancy to be fine-tuned in water, compensating for potential density differences between the virtual water and the actual water in the experimental settings. That is, each virtual model accounts for ~ 9 g of internal liquid, but the actual volume of this liquid can be adjusted in the physical robot with a syringe through a self-healing rubber valve (Table S1; Fig. 1).

The 3D position of the total center of mass was manipulated by accounting for the local centers of mass of each material of unique density. Materials like the batteries, motor, and electronic components were each assigned bulk density values because they are made up of composite materials. While this is an approximation, their contributions to the total center of mass are low because they account for small fractions of the total model mass (Tables S1 and S2). These components, like all others, were digitally modeled in Blender^77^ and their volumes and centers of mass were computed in the program MeshLab^76^. A dense, bismuth counterweight was also modeled, and positioned to artificially stabilize each model (pulling the z component of the total center of mass downward, while maintaining the horizontal components). The virtual model of this counterweight was used to make a 3D-printed mold, allowing a high heat silicone mold to be casted. The bismuth counterweight was cast from this silicone mold and filed/sanded to the dimensions of its virtual counterpart. Hyponomes were oriented horizontally, to yield movement in this direction. To maintain the same static orientation as the virtual model (same x and y center of mass components), the PETG center of mass was computed with the following equation:

4 $$D_{PETG} = \frac{{M\mathop \sum \nolimits_{i = 1}^{n} m_{i} - \mathop \sum \nolimits_{i = 1}^{n} (D_{i} m_{i} )}}{{\left( {m_{PETG} } \right)}}$$

where D_PETG_ is the location of the PETG center of mass from an arbitrary datum in each principal direction. M is the total center of mass in a particular principal direction, m_i_ is the mass of each model component, D_i_ is the local center of mass of each model component in a particular principal direction and m_PETG_ is the mass of the PETG required for a neutrally buoyant condition. See Tables S1 and S2 for a list of model components and measurements.

Each model was 3D printed with an Ultimaker S5 3D printer using clear (natural) PETG in separate parts, allowing the internal components to be implanted (i.e., brushed DC motors and bismuth counterweights). Each model part was chemically welded together with 100% dichloromethane, with minor amounts of cyanoacrylate glue used to fill seams (e.g., the water pump lid; Fig. 10a). Each final model consists of the main body (housing the water pump, motor, and counterweight), and a “lid” with brackets that house the electronics cartridge (Figs. 2 and 10). The main body and lid were fused together before each experiment by placing wax (paraffin-beeswax blend) along a tongue and groove seam, heating it with a hairdryer, then vigorously squeezing each part together. Surplus wax extruded from the seam was removed and smoothed, producing a water-tight seal.

### Thrust calibration

Even though each model was designed to have equal mantle cavity and pump cavity volumes, they produced slightly different thrusts. These differences were likely due to variable degrees of friction between the impellers and the surrounding water pumps. To correct for these differences, the thrust produced by each model was measured with a Vernier Dual-Range Force Sensor (0.01 N resolution). Each robot was attached at the hyponome location, through a series of pulleys, and to the sensor with fishing line (Fig. S1; similar to the methods used for living cephalopods^78^). Force was recorded for 30-s intervals at a sample rate of 0.05 s. During this time, each model was recorded jetting with a 6-s pulse for 15 trials (Fig. S2A). Each trial had initial noise from setting up the model, then peaked randomly when the fishing line became taught, then stabilized after some period of oscillation. Only the stabilized portion of the thrust profile was used to record thrust at 100% voltage for each model (Fig. S2B). The true zero datum was also subtracted from each of these trials. The lowest thrust from each of the models was used as a baseline (serpenticone and oxycone). Each model was recorded again for 15 trials by lowering the motor voltage in increments of 5% until they yielded similar thrusts (0.3 N) to the original serpenticone and oxycone trials (Fig. S2C). The final power levels were then determined for each model and adjusted with pulse-width modulation (PWM) through the microcontroller: serpenticone (100%), oxycone (100%), sphaerocone (95%), and morphospace center (85%).

The peak thrust measured for 1 kg extant *Nautilus* is around 2 N^16^. The time-averaged thrust during each pulse is around 23% of this value (0.46 N^16^). This computed value slightly overpredicts observed maximum velocities for this animal (33 cm/s instead of 25 cm/s), so the appropriate time-averaged thrust is probably slightly lower. The motor in the robots quickly reaches its maximum thrust (~ 0.3 N) once initiated then quickly declines after shutting off (Fig. S2). Therefore, the thrust produced by the robots can be treated as a conservative *Nautilus*-like jet thrust close to the behavior of escape jetting. One-second pulse and refill intervals are also on par with values reported for extant *Nautilus*^16^.

### Robot buoyancy

Each of the models were made near neutrally buoyant by adjusting the allotted ~ 9 g of internal liquid with a syringe through a self-healing rubber valve. The single-pulse experiments were performed in an external pool (ranging ~ 23.5 to 26.5 °C). The three-pulse and maneuverability experiments were performed in an internal pool (the Crimson Lagoon at the University of Utah). This internal pool had slightly higher temperatures (~ 28 °C), yielding lower ambient water densities than the virtual water. These conditions required slightly less internal liquid (~ 2–5 g). These differences in internal liquid masses produced negligibly small shifts in mass distributions because they are very small proportions of total robot masses (Table S1).

Perfect neutral buoyancy cannot be practically achieved, but this condition can be closely approached. Each of the biomimetic robots experience subtle upward or downward movements of the course of their 5–15 s long trials due to slightly positive or negative buoyancies. Because these differences in buoyancy influence the vertical component of movement, only the horizontal components are considered for discussion. However, a comparison of velocities computed from full, 3D movement (Eq. 5) and restricted 2D components (Eq. 6) reveals that these differences are minor (Figs. S7 and S8). These comparisons demonstrate that model buoyancy did not substantially influence kinematics other than gross trajectories (Figs. 4 and S9).

### 3D motion tracking

After adjusting buoyancy, each model was positioned underwater with a grabber tool. This tool was fitted with a bundle of fiber-optic cable (Fig. S4) attached to an infrared remote control. Arduino code (Dataset S2) was uploaded to the microcontroller in the robot allowing jet pulse duration, number of pulses, and power to be adjusted with this remote control. After an infrared pulse is received, the motor activates, and activity is indicated by a green LED that illuminates the model from the inside. This light is used to determine time-zero for each trial of motion tracking.

After sending an infrared signal, the movement of each model was recorded with a submersible camera rig fitted with two waterproof cameras (Fig. 3). Each of the four models were monitored during a single, one-second jet for at least 9 trials each. Additionally, the laterally compressed morphotypes (serpenticone and oxycone) were monitored during three, one-second pulses for 10 trials each. The inflated morphotypes (sphaerocone and morphospace center) were not able to be monitored over longer distances because they had the tendency to rotate about the vertical axis, obscuring views of the tracking points. In addition to horizontal movement, turning efficiency (maneuverability about the vertical axis) was monitored by directing the cameras with a top-down view of each model. A 90° elbow attachment for the hyponome was fit to each model to investigate the ease or difficulty of rotation. Each model was designed to spin counter-clockwise when viewed from above so that the influence of the motor’s angular momentum was consistent between models.

Footage was recorded with two GoPro Hero 8 Black cameras at 4K resolution and 24 (23.975) frames per second, with linear fields of view. Motion tracking was performed with the software DLTdv8^79^ to record the pixel locations of each tracking point (Figs. 1c and S4). These coordinates were transformed into 3D coordinates in meters using the program easyWand5^80^. The tracking points on each model were used for wand calibration because the distances between these sets of points were fixed. Standard deviations of the reproduced tracking point distances of less than 1 cm were considered suitable.

The 3D position datasets allowed velocity, acceleration, rocking, to be computed for each experiment. Additionally angular displacement and angular velocity was of interest for the rotation experiments about the vertical axis. Velocity was computed under two scenarios: (1) using the 3D movement direction between each timestep (Eq. 5), and (2) only considering the horizontal movement direction between each time step (Eq. 6). The latter scenario was preferred to nullify the influences of model buoyancies, which were not perfectly neutral and caused some degree of vertical movement.

5 $$V_{i} = \frac{{\sqrt {\left( {x_{i} - x_{i - 1} } \right)^{2} + \left( {y_{i} - y_{i - 1} } \right)^{2} + \left( {z_{i} - z_{i - 1} } \right)^{2} } }}{{\left( {t_{i} - t_{i - 1} } \right)}}$$

6 $$V_{i} = \frac{{\sqrt {\left( {x_{i} - x_{i - 1} } \right)^{2} + \left( {y_{i} - y_{i - 1} } \right)^{2} } }}{{\left( {t_{i} - t_{i - 1} } \right)}}$$

where V and t are velocity and time, and the subscripts i and i −1 refer to the current and previous time steps, respectively. Coordinate components are denoted by x, y, and z at each timestep. The averaged 3D location of both tracking points was used for each model (i.e., midpoints). Note that Eq. (5) uses the 3D form of the Theorem of Pythagoras, whereas Eq. (6) uses the 2D version. Time zero for each trial was defined as the frame where the robot was illuminated by the internal LED, indicating motor activity. Acceleration was modeled by fitting a linear equation to the datapoints during the one-second pulse interval(s) using the curve fitting toolbox in MATLAB R2020A.

The artificially high hydrostatic stability of each model was designed to nullify rocking during movement. This behavior was computed for each model during the one-pulse experiments with the following equation:

7 $$\theta_{dv} = \cos^{ - 1} \left( {\frac{{\left( {z_{2} - z_{1} } \right)}}{{\sqrt {\left( {x_{2} - x_{1} } \right)^{2} + \left( {y_{2} - y_{1} } \right)^{2} + \left( {z_{2} - z_{1} } \right)^{2} } }}} \right) - \theta_{tp}$$

where $\theta_{dv}$ is the angle deviated from true vertical and $\theta_{tp}$ is the angle of the tracking points measured from the vertical in a static setting. The subscripts 1 and 2 of the x, y, and z coordinates refer to the anterior and posterior tracking points, respectively.

Maneuverability about the vertical axis was determined by computing the angle between the horizontal components of each tracking point. The net angle from the starting angle for each trial was tabulated. Angular velocity was determined by dividing the change in angle between each frame by the frame duration (1/23.975 fps).

Links to example motion tracking footage, and robotic models are deposited in an online repository^60,61,63^ (Dataset S2; https://doi.org/10.5281/zenodo.6180801).

## Supplementary Information

Supplementary Information.

## Data Availability

Virtual models of the theoretical planispiral cephalopods and biomimetic robots are available in Datasets S1 and S2, along with sample footage and Arduino code (https://doi.org/10.5281/zenodo.5684906; https://doi.org/10.5281/zenodo.6180801; see Supplementary information).
