# Supplementary material for: Resurrecting extinct cephalopods with biomimetic robots to explore hydrodynamic stability, maneuverability, and physical constraints on life habits
Source: Sci Rep. 2022 Jul 4;12:11287. doi: 10.1038/s41598-022-13006-6 (PMC9253093; doi:10.1038/s41598-022-13006-6)
Supplement: Supplementary file 1 — Supplementary Information. [file 41598_2022_13006_MOESM1_ESM.pdf]

Supplementary materials for

Resurrecting extinct cephalopods with biomimetic robots to explore hydrodynamic stability, maneuverability, and physical constraints on life habits

David J. Peterman and Kathleen A. Ritterbush

*Corresponding author email: David.Peterman@utah.edu

This PDF file includes

Supplementary text

Figures S1 to S14

Tables S1 to S17

Legend for Datasets S1 and S2

SI References

Supplementary Text

Measurements of septal spacing were recorded from a CT-scanned *Nautilus pompilius* conch. These measurements were recorded as the angle between the ventral edge of the current and previous septum and the spiraling axis of the shell. Because septal spacing differs in early ontogeny (Fig. S11), only measurements from the 7th to 33rd (terminal) septum were considered. The average angle of 23.46° ± 3.32° (standard deviation) was rounded to 23° and held constant through the ontogeny of the hydrostatic models.

Measurements of shell and septum thickness were recorded as a ratio of inner whorl height (Table S13) in order to apply thickness to the theoretical planispiral models. These models were created with array instructions (Table S14) in Blender (Blender Online Community) to build the shell from measurements of representative specimens (*Sphenodiscus* – oxycone; *Dactylioceras* – serpenticone; and *Goniatites* – sphaerocone).

The whorl section of the center model is an average (Fig. S12) of the near-endmember models (oxycone, serpenticone, and sphaerocone).

Westermann morphospace parameters were computed from measurements on each virtual model (Fig. S13; Table S15). These parameters fall on a ternary diagram with variable whorl expansion (w), umbilical exposure (U), and inflation (i.e., thickness ratio; Th). Each are computed with the equation below with variables corresponding to those depicted in Figure S13:

$$w = a/a' \quad \text{(S1)}$$
$$U = UD/D \quad \text{(S2)}$$
$$Th = b/D \quad \text{(S3)}$$

The peripheral shape of the *Nautilus* septum (suture line) and its general first-order morphology was preserved while modifying it to fit within the whorl sections of the theoretical models (Fig. S14).

Calculation of drag coefficients (C_d)

Drag coefficients do not capture the hydrodynamics of a single shape moving at different velocities (or with changing scale; i.e., Reynolds numbers). However, these coefficients can be used to approximate the relative differences in hydrodynamic drag between different morphologies. Drag coefficients were computed from analyzing the deceleration of each biomimetic robot during the one-second pulse experiments after they ceased jetting (i.e., as they fell from their maximum attained velocities). These coefficients were determined in MATLAB by creating a modeled velocity function that reduced the sum of squared error between this function and the data points computed from all trials for each examined morphology. The velocity function (Equation S5) is derived from the drag force equation (Equation S4) and follows the form:

$$F_d = 0.5V\text{\^{}}(2/3)\rho C_d u^2 \quad \text{(S4)}$$

$$u(t) = \frac{mu_0}{0.5V^{(2/3)}\rho C_d u_0(t-1)+m} \quad \text{(S5)}$$

Where $u(t)$ is the velocity as a function of time (t), m is mass, u_0 is the initial velocity, V is volume (note that this becomes equivalent to the area term, and that volume is nearly identical between each robot), ρ is density, C_d is the drag coefficient. Drag coefficients were fit with the curve fitting toolbox in MATLAB. Note that the time term in the equation has 1 second subtracted. This value corrects for the time the motor was active and translates the curve to the moment the robots stopped jetting. Drag coefficients and modeled velocity function terms are reported in Fig. S6 and Table S5.

Calculation of moments of inertia

Moments of inertia for each material of unique density for the robots (PETG thermoplastic, electronics cartridge, bismuth counterweight, pump and chamber liquid, motor (bulk ρ), both batteries (bulk ρ), electronics (bulk ρ), and self-healing rubber) and the virtual hydrostatic models representing the living animals (soft body and shell) were computed to determine their total moments of inertia (Table S10; sum of each component). The moments of inertia for each individual component were computed with the program MeshLab. The relative importance of the moments of inertia versus hydrodynamic effects (drag and wake dynamics, etc.) were determined by comparing the angular velocities computed for rotation in a vacuum (after one second resulting from 0.3N of thrust) and those observed after one second of jetting in the robots. Angular velocities (ω) in a vacuum were computed with the following equations:

$$\tau = Fr = I\alpha \quad \text{(S6)}$$

$$Fr/I = \alpha = (\omega_f - \omega_i)\Delta t \quad \text{(S7)}$$

Where τ is torque, F is the force of thrust (~0.3N for the robots), r is the length of the lever arm measured between the source of jet thrust (i.e., the hyponome) and the vertical axis passing through the centers of buoyancy and mass (measured from the virtual models in the program Blender; Table S11), I is the total moment of inertia (Table S10), and α is angular acceleration. ω_f and ω_i are angular velocities after one second of jetting and just before jetting (equal to zero), respectively, and Δt is the change in time (equal to one second).

Potential differences in rotational kinematics due to different moments of inertia between the robots and the virtual hydrostatic models (representing theoretical morphologies of the living animals) were estimated by comparing the proportion of the observed ω values after one second of jetting, and the ω values computed from each total moment of inertia value (Table S11).

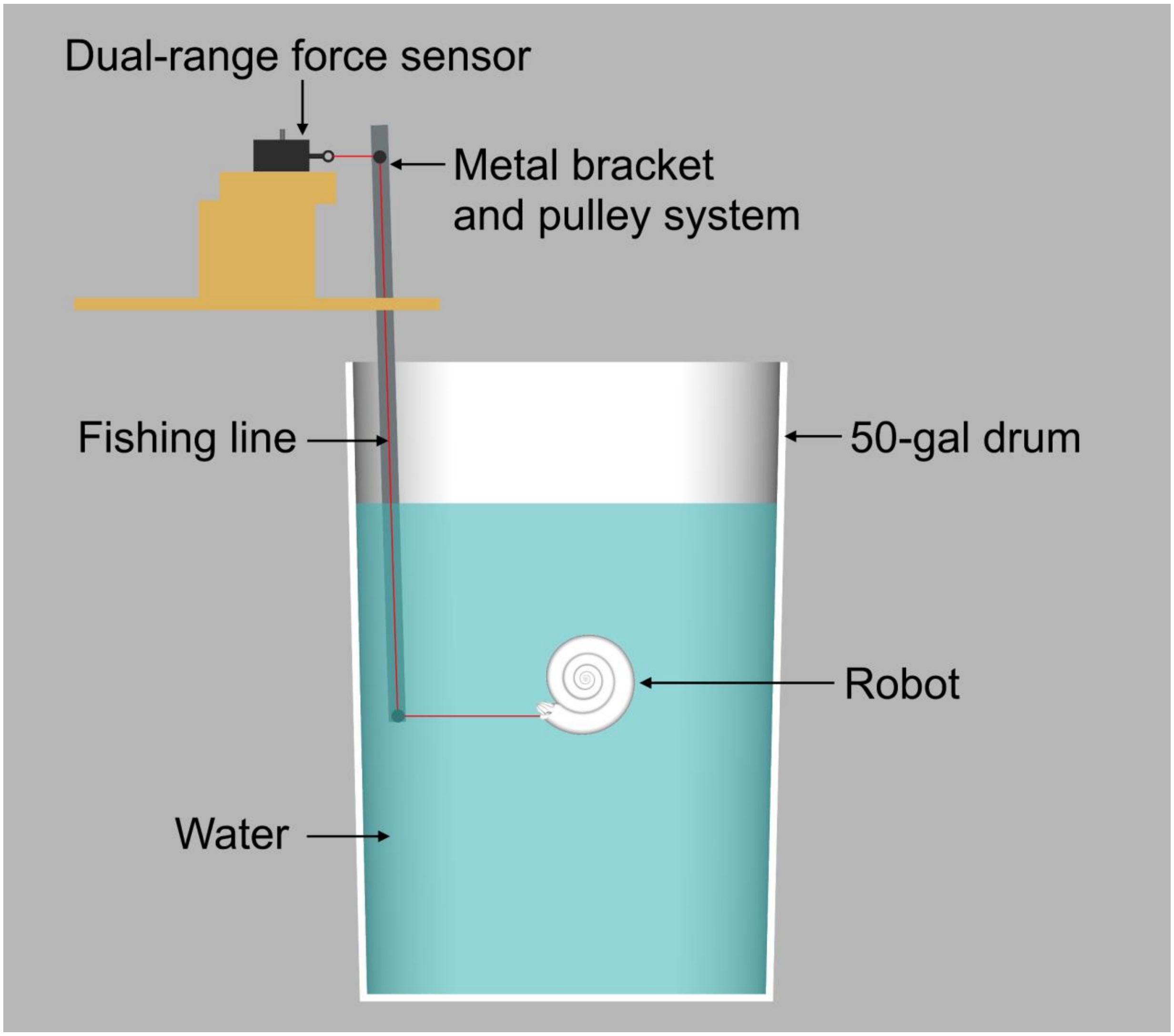

Figure S1: Schematic of the thrust calibration setup. Each robot was placed in water with a fishing line attached from the hyponome location, through a series of pulleys, to a dual-range force sensor. Rendered in MeshLab.

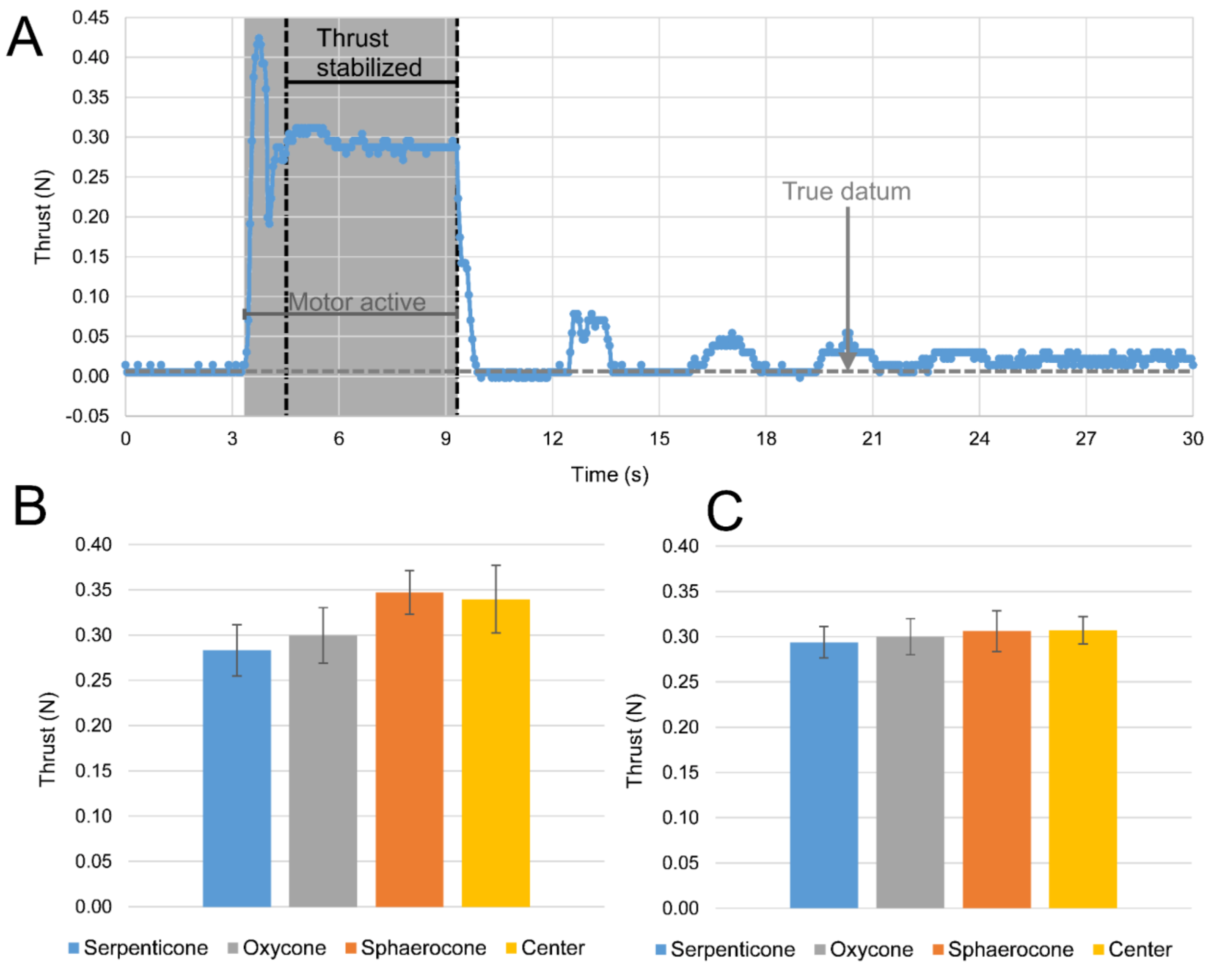

Figure S2: Thrust calibration of biomimetic cephalopod robots. A) Example of a single recorded trial. Force was measured for 30 seconds to capture the 6-second region of motor activity (highlighted grey). The model pulled a fishing line on a pulley system, attached to the force sensor. When the line became taught, force peaked, then oscillated. Only the stable region (denoted between the two dashed lines) was used to measure the average thrust for each trial. After isolating this region, the true zero-datum was subtracted from the data series. B) Average thrust recorded during 15 trials for each model with the motor running at 100% maximum voltage (7.4V). C) Average thrust recorded during 15 trials for each model with voltage adjusted via pulse-width modulation (Serpenticone = 100%; Oxycone = 100%; Sphaerocone = 95%; Center = 85%. Error bars represent one standard deviation.

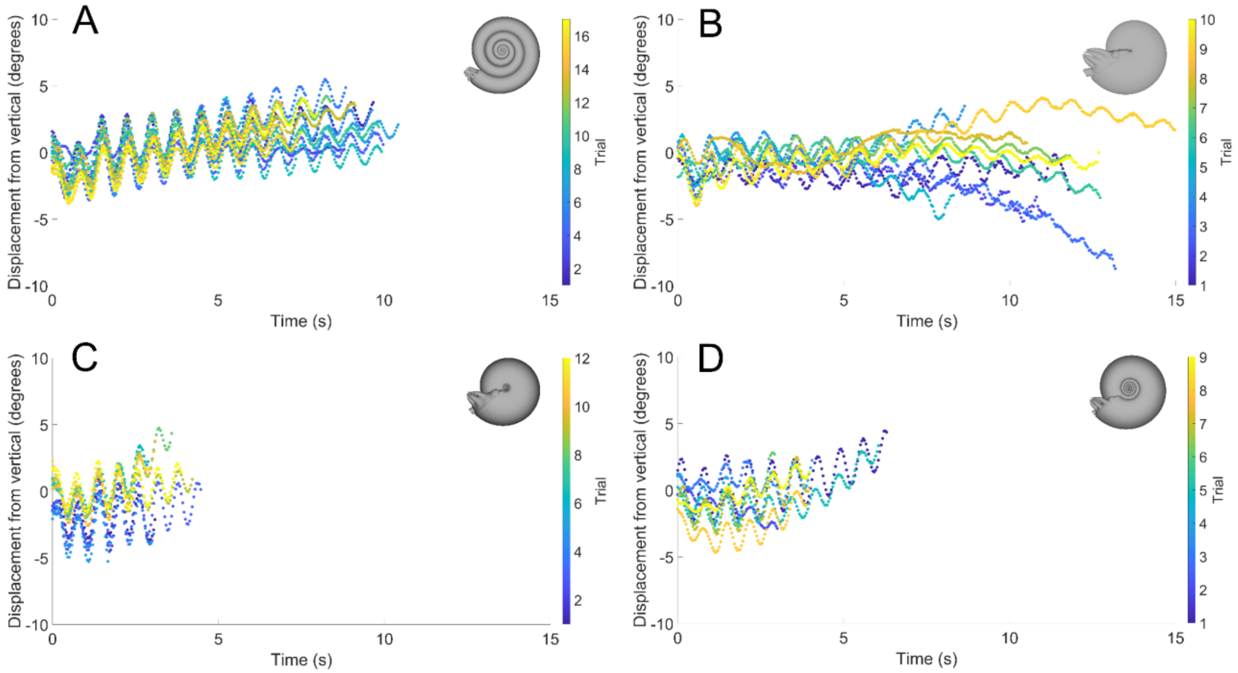

Figure S3: Rocking during movement demonstrated by computing the angle displaced from the static orientation. A) Serpenticone, B) oxycone, C) sphaerocone, and D) morphospace center. These models with artificially high hydrostatic stability, experience minimal rocking (+/- 5 degrees).

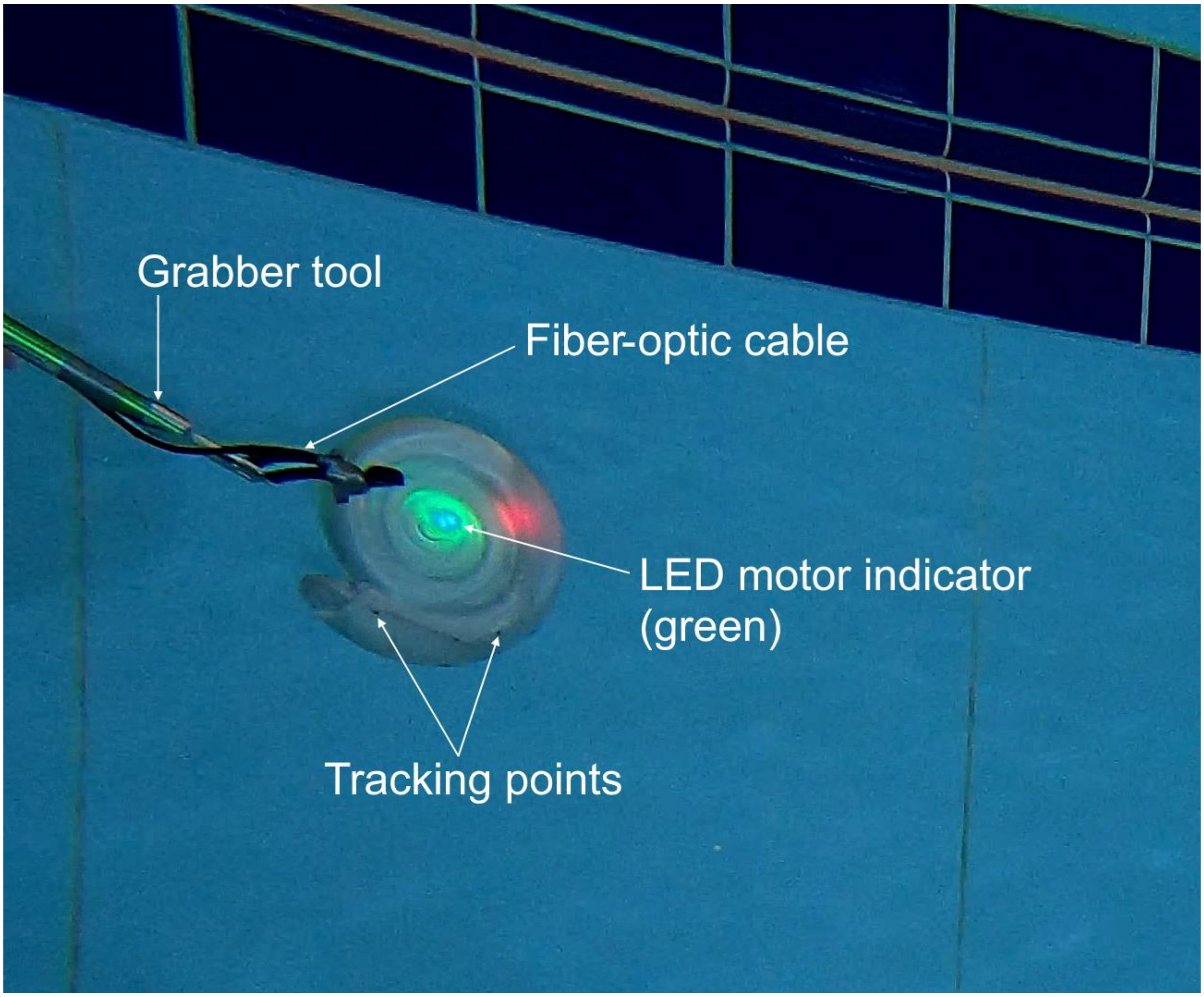

Figure S4: Example frame of the motion tracking footage showing grabber tool used to position the biomimetic robot. After releasing the robot, a fiber optic cable was used to deliver an infrared pulse that initated the motor. At the same time, a green indicator LED illuminated the model while the motor was active. This light was used to determine time-zero for each experiment. Various kinematics were monitored with the two tracking points placed on each model.

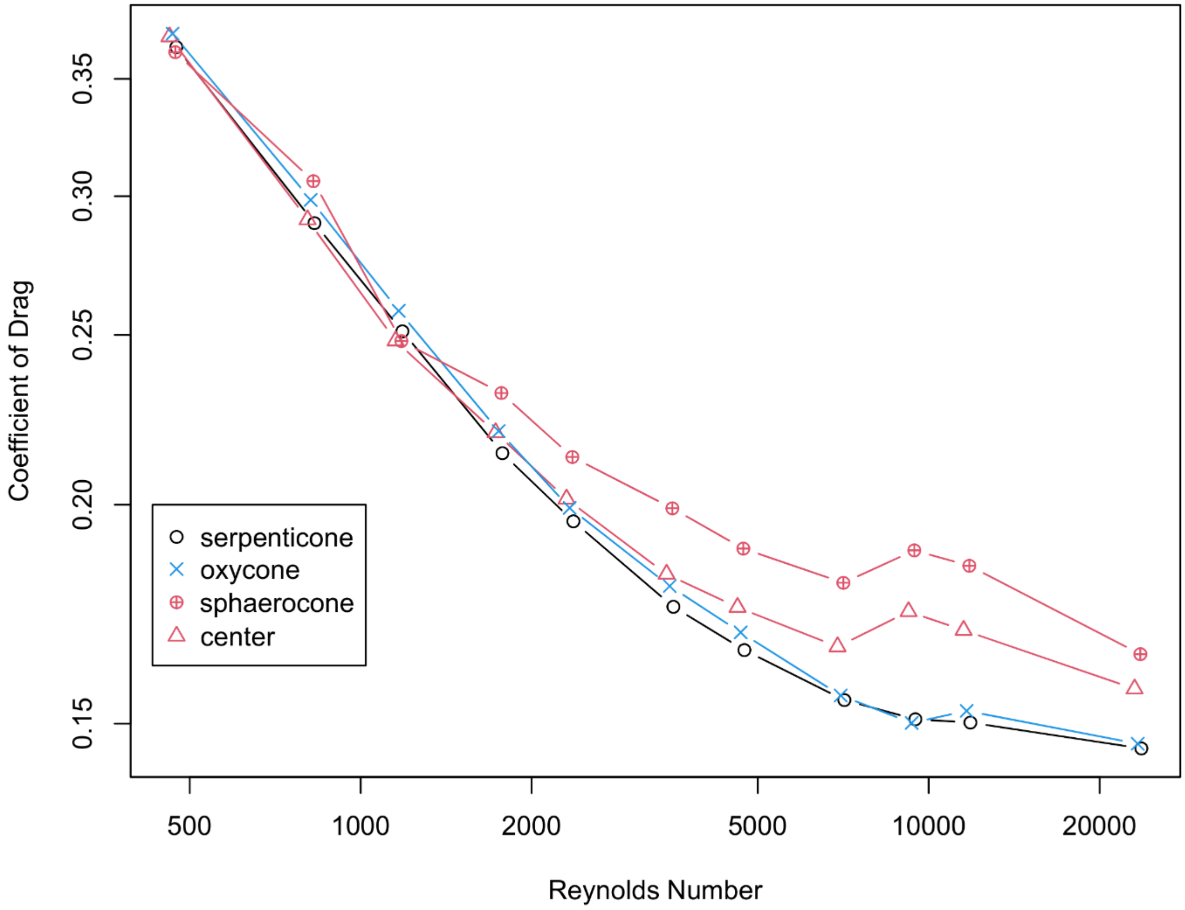

Figure S5: Example of drag coefficient and Reynolds number curves for conch shapes similar to the cephalopod robots. Since drag coefficients vary based on size and swimming speed, hydrodynamic properties are interpreted in terms of velocity and acceleration.

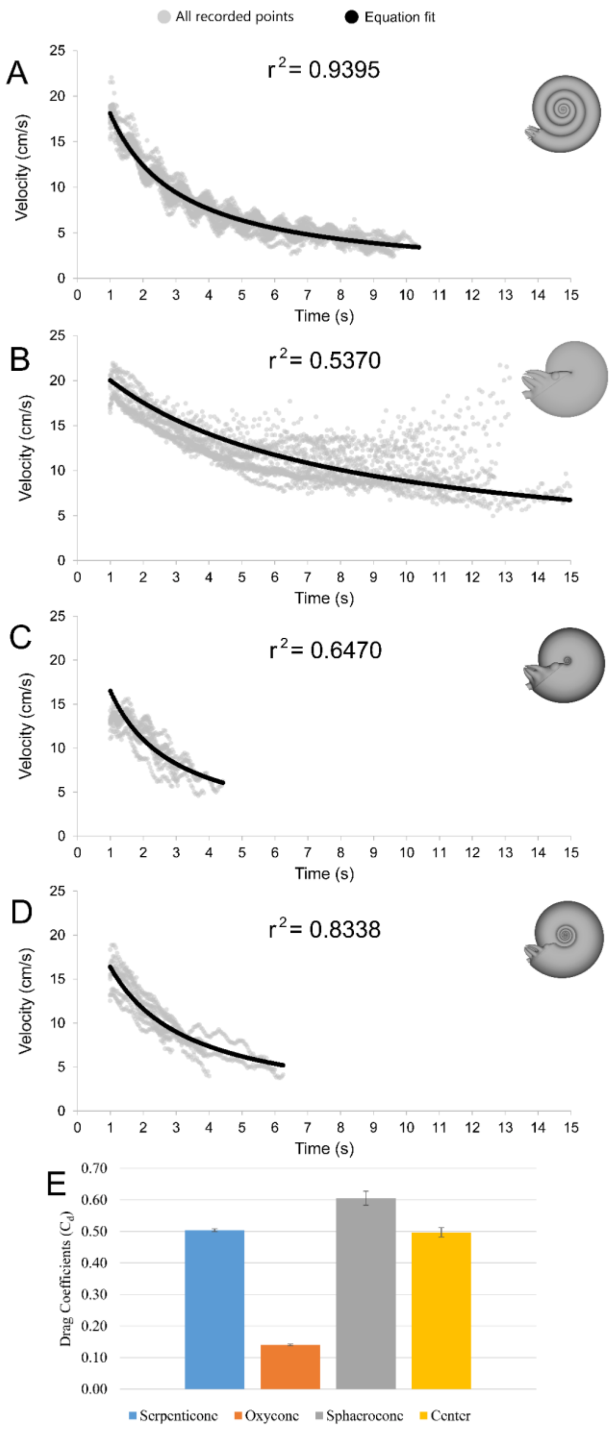

Figure S6: Analysis of drag coefficients for the serpenticone (A), oxycone (B), sphaerocone (C), and morphospace center (D). Drag coefficients (E) were determined by minimizing the sum of squared errors between the modeled velocity function (black) and all computed datapoints for each morphotype. The errors bars on panel E denote 95% confidence intervals for the drag coefficients. Note that the serpenticone and sphaerocone cannot be statistically distinguished.

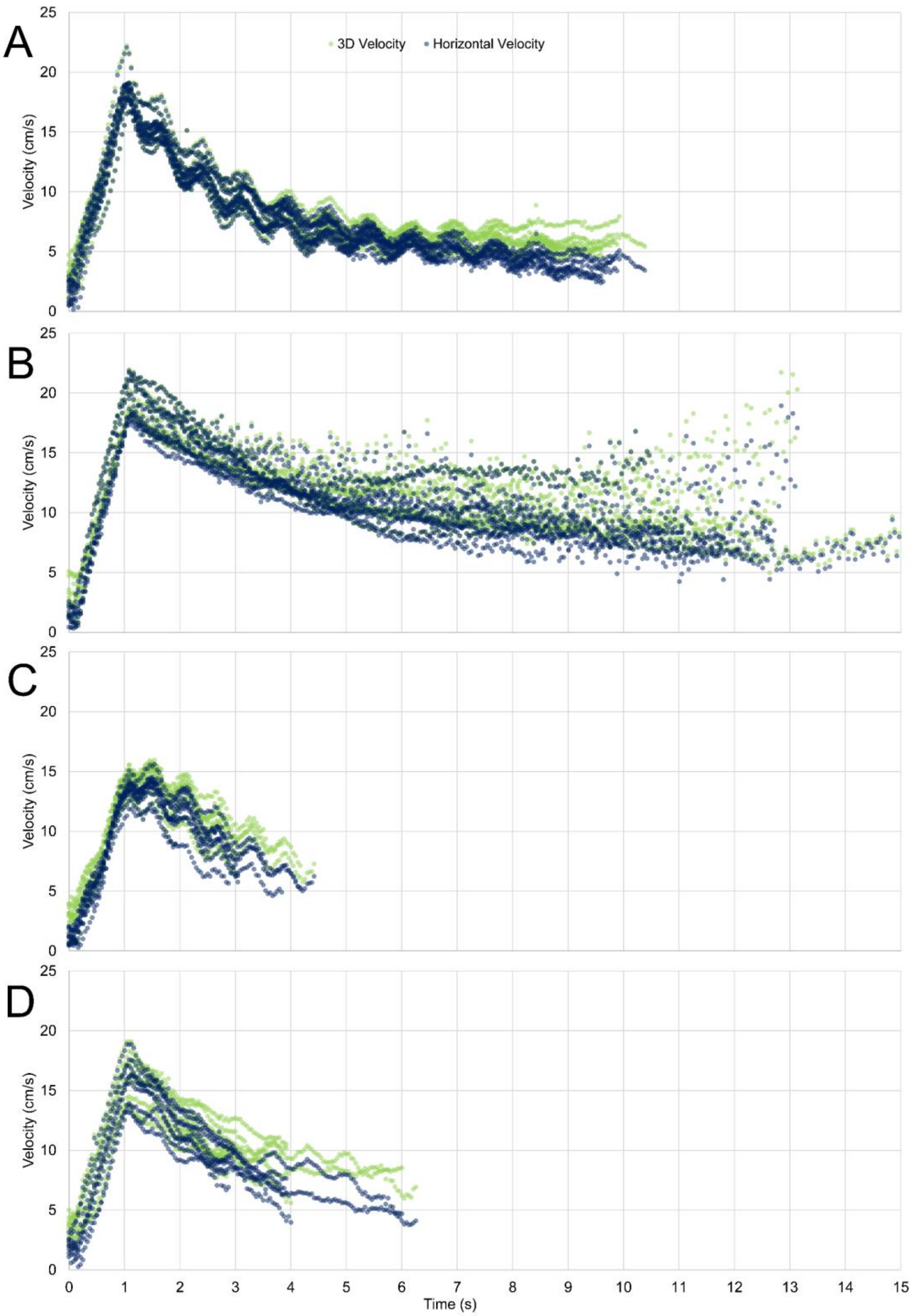

Figure S7: Comparison of velocity computed from movement in any 3D direction (green) and only the horizontal component of velocity (blue). A) Oxycone, B) serpenticone, C) sphaerocone, and D) morphospace center. Velocity was analyzed in response to a single, one-second jet pulse. Horizontal velocity is preferrred because it ignores vertical movement caused by subtle deviations from neutral buoyancy.

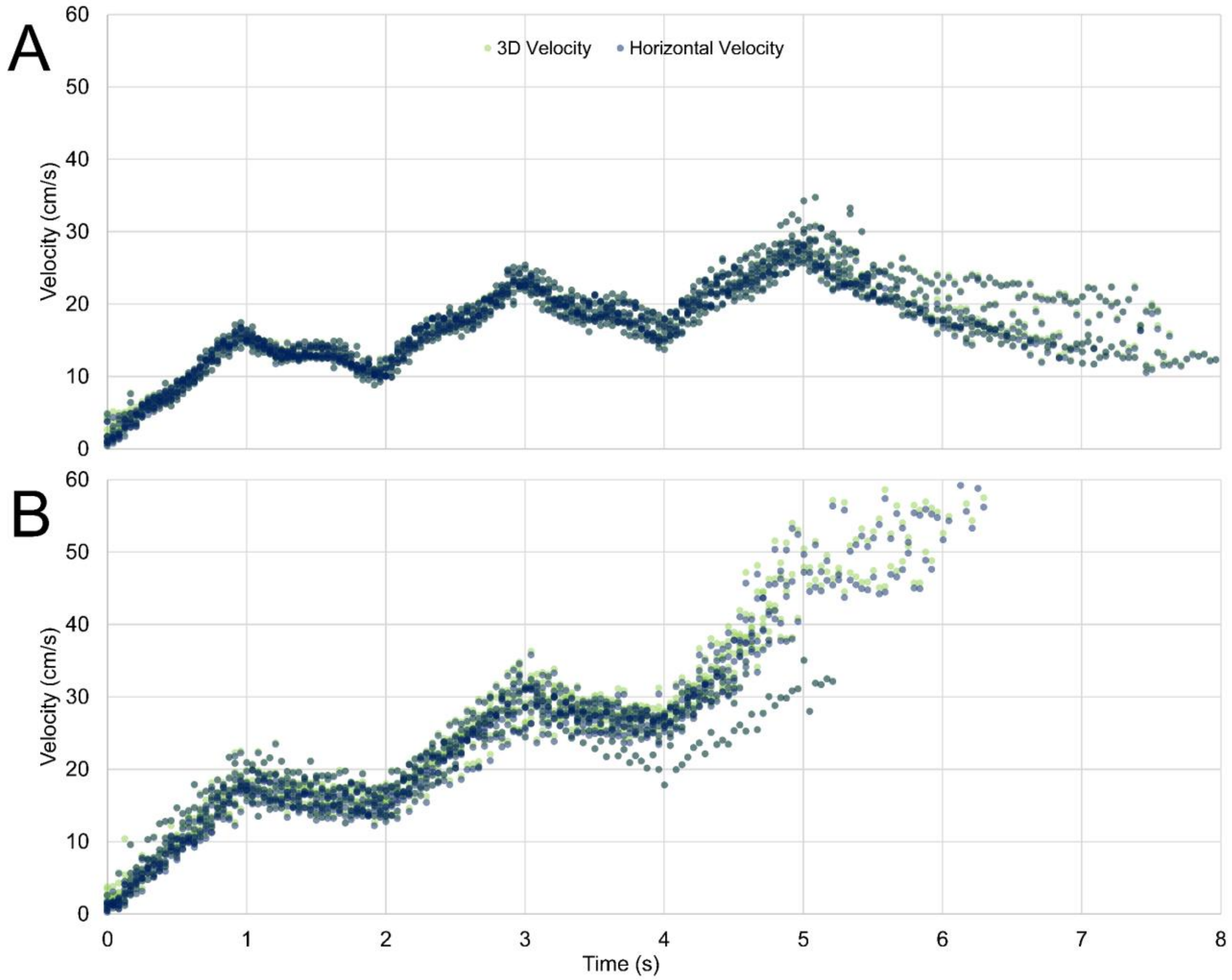

Figure S8: Comparison of velocity computed from movement in any 3D direction (green) and only the horizontal component of velocity (blue). A) Oxycone, B) serpenticone, C) sphaerocone, and D) morphospace center. Velocity was analyzed in response to three, one-second jet pulses.

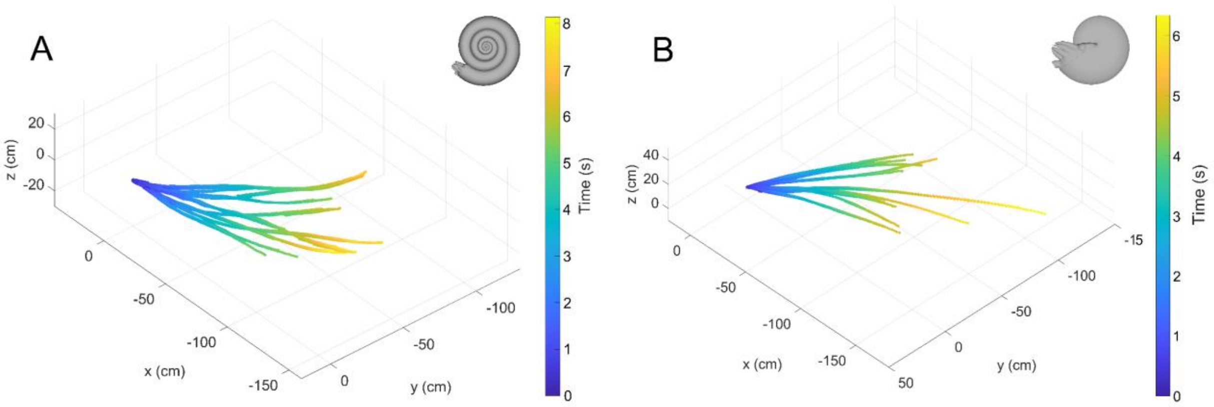

Figure S9: 3D position of the serpenticone robot (A) and oxycone robot (B) during three, one-second jet pulses.

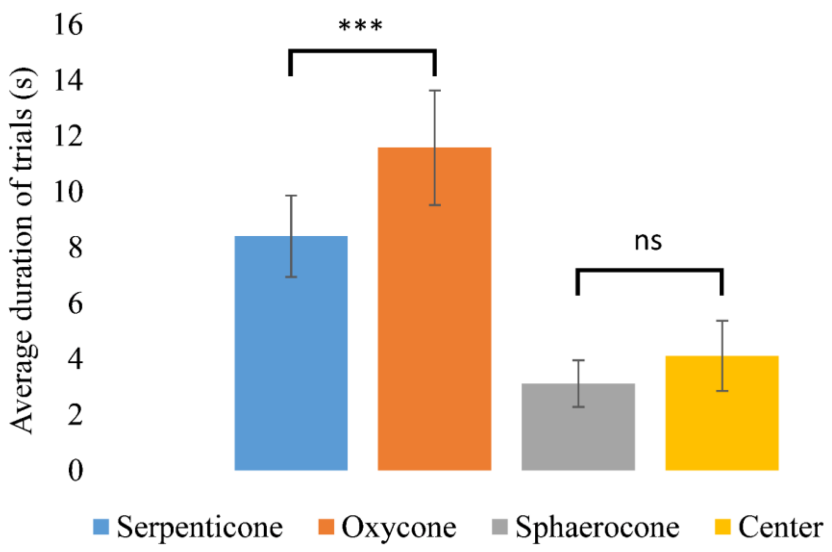

Figure S10: Average duration of all trials for the serpenticone (n = 17), oxycone (n = 10), sphaerocone (n = 12), and morphospace center (n = 9). Error bars represent standard deviations. A one-way ANOVA with a Games-Howell post hoc test was used to distinguish means. Note that the differences of all other combinations than those denoted are significant at the $p \leq 0.0001$ level.

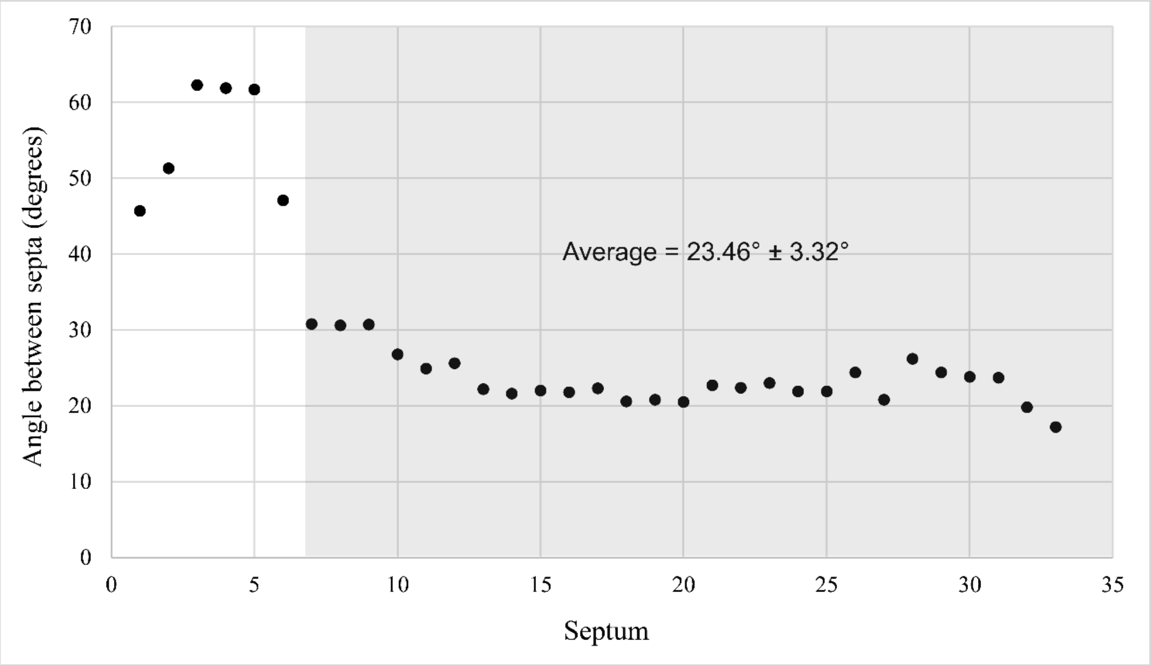

Figure S11: Septal spacing for each septum recorded from a CT-scanned conch of *Nautilus pompilius*. The shaded region corresponds to the angles that were considered for the theoretical conch models (7th to 33rd septum).

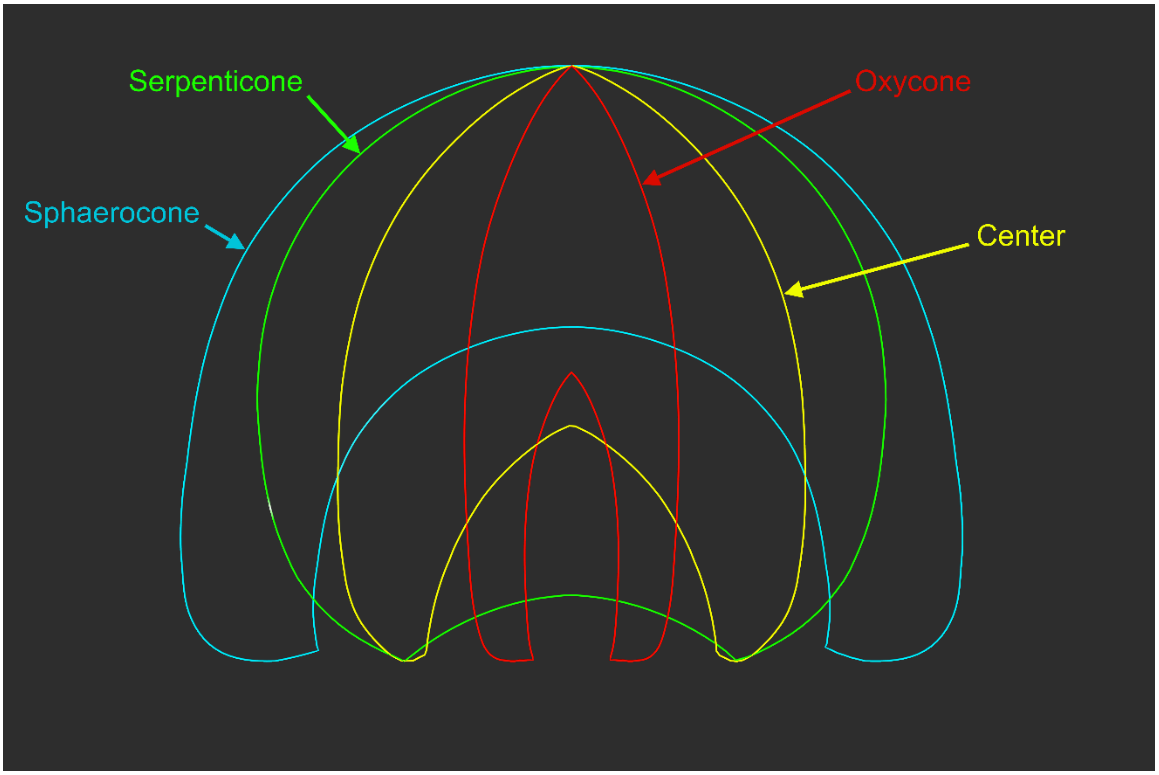

Figure S12: Whorl sections of each near end-member model, averaged to produce the morphospace center whorl section. Rendered in Blender.

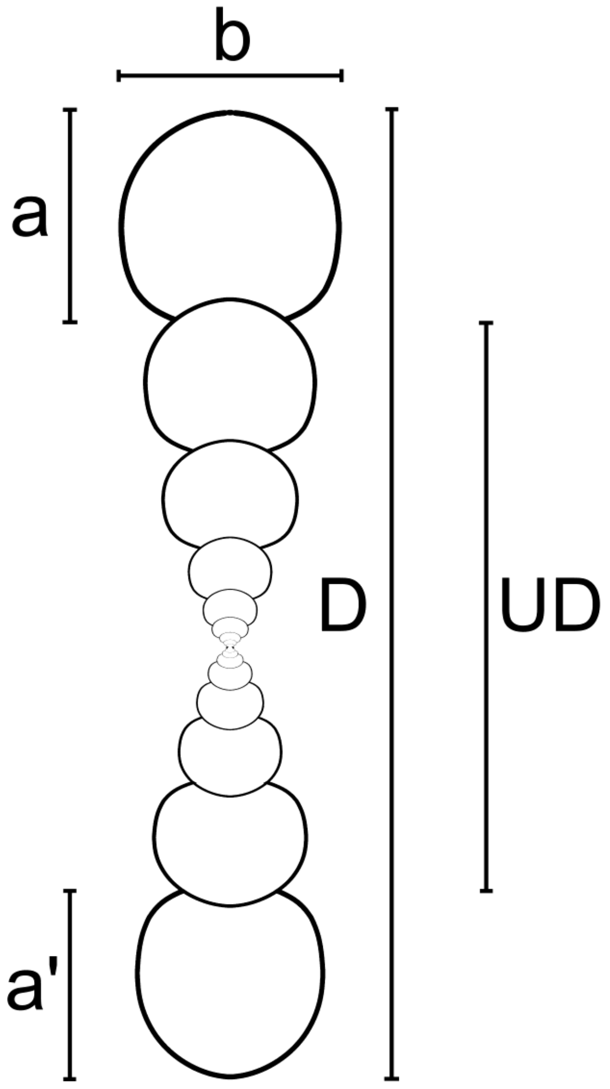

Figure S13: Transverse view of the serpenticone model with conch measurements used to compute Westermann Morphospace parameters (*1*).

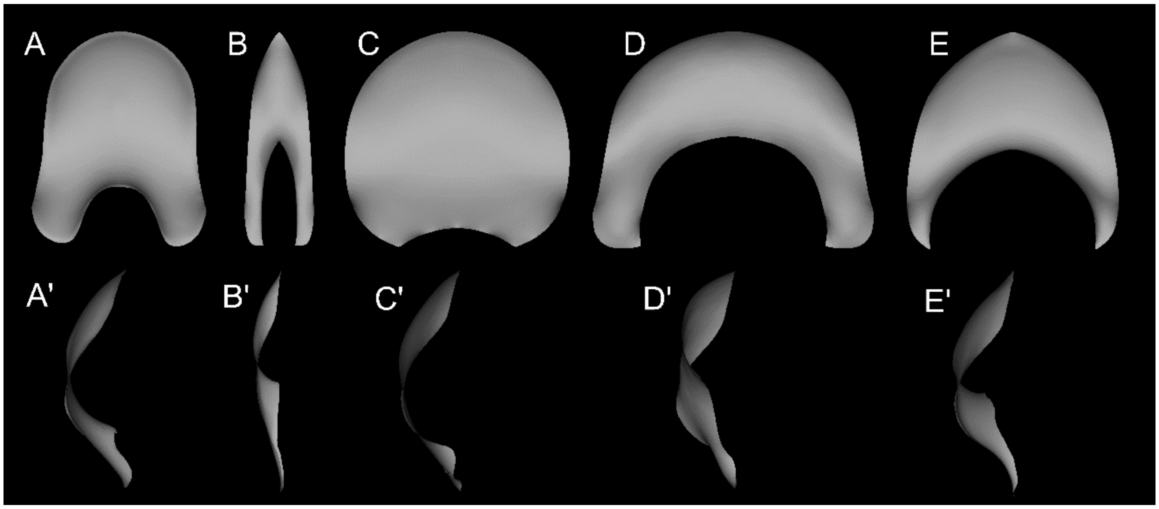

Figure S14: Adoral views of septa derived from the *Nautilus pompilius* CT scan: *Nautilus pompilius* (A), oxycone (B), serpenticone (C), sphaerocone (D), and the morphospace center (E). Prime symbols denote side views of each respective septum. Rendered in MeshLab.

Table S1: Densities, volumes, and masses of each biomimetic robot component.

		Serpenticone		Oxycone		Sphaerocone		Center	
**Model component**	**Density (g/cm^3)**	**Volume (cm^3)**	**Mass (g)**	**Volume (cm^3)**	**Mass (g)**	**Volume (cm^3)**	**Mass (g)**	**Volume (cm^3)**	**Mass (g)**
**PETG**	1.264	386.705	488.795	412.539	521.449	361.987	457.552	393.740	497.688
**Electronics cartridge**	1.280	18.690	23.930	18.690	23.930	18.690	23.930	18.690	23.930
**Bismuth counterweight**	9.445	39.285	371.055	37.099	350.404	43.139	407.456	38.532	363.937
**Liquid**	1.000	8.837	8.839	9.156	9.158	8.594	8.596	8.729	8.731
**Motor**	2.448	6.754	16.537	6.754	16.537	6.754	16.537	6.754	16.537
**3.7V battery**	2.054	3.749	7.700	3.749	7.700	3.749	7.700	3.749	7.700
**7.4V battery**	2.013	15.828	31.865	15.828	31.865	15.828	31.865	15.828	31.865
**Electronics**	1.674	8.831	14.785	8.831	14.785	8.831	14.785	8.831	14.785
**Self-healing rubber**	1.568	0.181	0.284	0.150	0.235	0.181	0.284	0.176	0.275
**Total**	~1.0002	971.686	971.880	981.680	981.876	972.211	972.405	971.678	971.872

Table S2: 3D positions of the local centers of mass for each biomimetic robot component, centers of buoyancy, and total centers of mass for each robot.

	Serpenticone			Oxycone			Sphaerocone			Center		
**Model component**	**x (mm)**	**y (mm)**	**z (mm)**	**x (mm)**	**y (mm)**	**z (mm)**	**x (mm)**	**y (mm)**	**z (mm)**	**x (mm)**	**y (mm)**	**z (mm)**
**PETG**	-0.2089	-87.4398	-22.7700	0.0235	-60.6790	13.5120	0.6231	-38.7902	8.5772	-0.0004	-55.0724	9.1587
**Electronics cartridge**	1.3819	-155.1883	2.5170	0.0160	-110.4815	46.4711	1.3819	-65.7878	44.8644	0.0160	-97.0811	42.2857
**Bismuth counterweight**	-0.0239	-84.2228	-83.5671	-0.0296	-45.5083	-50.7937	-0.8974	-51.4005	-21.5804	0.0000	-57.4218	-31.7879
**Liquid**	-0.1315	-36.6280	-76.9985	-0.1296	2.6512	-30.5089	-0.1321	-16.3298	-22.0300	-0.1339	-14.2594	-17.6778
**Motor**	-0.0724	-36.6293	-55.6100	-0.0724	4.5616	-4.5735	-0.0724	-19.7523	-2.5171	-0.0724	-14.8913	4.7590
**3.7V battery**	1.6933	-148.9236	32.1315	0.3274	-96.8907	73.5183	1.6933	-43.4312	65.2714	0.3274	-84.0004	69.5833
**7.4V battery**	3.1394	-151.3800	-19.7106	1.7736	-112.4518	24.0059	3.1394	-75.6537	24.5855	1.7736	-98.6292	19.7874
**Electronics**	-2.3145	-139.8525	-2.5153	-4.2723	-95.7804	42.0132	-3.7539	-53.7217	35.9625	-4.0831	-83.0123	38.1763
**Self-healing rubber**	0.0000	-84.9722	-104.0069	0.0000	-41.7995	-77.1246	0.0000	-54.9137	-41.6553	-0.0044	-57.6081	-53.2112
**Center of buoyancy**	-0.0019	-90.0625	-9.0821	-0.0043	-57.1940	19.3378	-0.0028	-45.7661	20.7011	-0.0045	-57.9794	22.2835
**Center of mass**	-0.0019	-90.0625	-45.7000	-0.0043	-57.1940	-8.2500	-0.0028	-45.7661	-2.3950	-0.0045	-57.9794	-4.5000

Table S3: Comparison of the distance between the centers of buoyancy and mass (BM) and hydrostatic stability index (St) between the biomimetic robots and virtual models. The robots have much higher stability, allowing the variable of shell shape to be isolated. Note these models have body lengths that result in *Nautilus*-like cameral liquid and have slightly higher stabilities than specimens with proper body chamber lengths.

	**Robots**		**Virtual models**	
**Model**	**BM (mm)**	**S_t**	**BM (mm)**	**S_t**
**Serpenticone**	36.618	0.370	3.380	0.034
**Oxycone**	27.588	0.278	10.366	0.104
**Sphaerocone**	23.096	0.233	1.686	0.017
**Center**	26.784	0.270	2.447	0.025

Table S4: Hydrostatic properties of each theoretical planispiral cephalopod. BCL = body chamber length measured in degrees, θ_a = static apertural orientation measured in degrees from vertical, θ_t = thrust angle measured in degrees from horizontal, Φ = the percentage of the phragmocone to be emptied of liquid for neutral buoyancy, S_t = hydrostatic stability index.

Model	BCL	θ_a	θ_t	Φ	S_t
Serpenticone	450	45.1	-32.2	92.5	0.034
Oxycone	150	53.1	-16.3	90.5	0.104
Sphaerocone	445	42.2	-33.7	90.9	0.017
Center	476	56.4	-20.3	84.5	0.025

Table S5: Drag coefficients (C_d) computed for each robot by analyzing the deceleration of all corresponding trials after they ceased jetting (i.e., falling from their maximum attained velocities). Each term in the modeled velocity function is reported below (m = mass, u_0 = initial velocity the moment jetting ceased, ρ = water density, V = volume which is raised to the 2/3 power to equal the area term, C_d = drag coefficient, Adj. r^2 = adjusted r-squared. Note that 95% upper and lower confidence intervals (CI+ and CI-, respectively) are listed for each computed drag coefficient. The serpenticone and center robots have overlapping confidence intervals, suggesting they cannot be statistically distinguished from each other.

	**Serpenticone**	**Oxycone**	**Sphaerocone**	**Center**
**m (Kg)**	0.9719	0.9819	0.9724	0.9719
**u_0 (m/s)**	0.1811	0.2004	0.1650	0.1639
**ρ (kg/m^3)**	1000	1000	1000	1000
**$V^{(2/3)}$ (m^2)**	0.009823	0.009891	0.009827	0.009823
**C_d**	0.5038	0.1401	0.6055	0.4968
**C_d CI+**	0.5081	0.1428	0.6277	0.5117
**C_d CI-**	0.4994	0.1373	0.5832	0.4819
**Adj. r^2**	0.9395	0.5370	0.6470	0.8338

Table S6: Acceleration (slope) and intercept of velocity increase in response to a single jet pulse for each biomimetic robot. Numbers in parentheses denote lower and upper 95% confidence intervals.

**Model**	**Acceleration (cm/s^2)**	**Intercept (cm/s)**	**R^2**
**Serpenticone**	16.77 (16.31, 17.23)	0.6887 (0.4209, 0.9564)	0.9242
**Oxycone**	17.98 (17.39, 18.57)	-0.1686 (-0.5124, 0.1751)	0.9359
**Sphaerocone**	12.68 (12.33, 13.03)	-0.03142 (-0.2351, 0.1723)	0.9450
**Center**	14.36 (13.58, 15.15)	0.4967 (0.03865, 0.9548)	0.8537

Table S7: Kinematic properties computed from the single-pulse, horizontal movement experiments. A one-way ANOVA with a Games-Howell post hoc test was used to distinguish differences between means. Green numbers = p-values < 0.05, red numbers = p-values > 0.05.

Model	Conch diameter (cm)	Ave. distance after 2.5 s (cm)	Ave. distance total (cm)	Peak velocity (cm/s)
**Serpenticone (n = 17)**	21.2	30.3 ± 2.2	71.71 ± 10.80	18.3 ± 1.3
**Oxycone (n = 10)**	23.5	34.9 ± 3.3	136.62 ± 22.95	19.5 ± 1.6
**Sphaerocone (n = 12)**	15	24.7 ± 2.2	32.13 ± 9.68	13.8 ± 0.9
**Center (n = 9)**	17.7	27.4 ± 3.6	44.09 ± 10.64	15.7 ± 1.9
**ANOVA (p values reported for each column)**				
**Serp v Oxy**	NA	7.11E-03	1.64E-05	2.64E-01
**Serp v Sphaero**	NA	2.16E-05	8.13E-10	4.75E-11
**Serp v Center**	NA	1.94E-01	5.09E-05	1.14E-02
**Oxy v Sphaero**	NA	2.93E-06	9.98E-08	9.84E-07
**Oxy v Center**	NA	1.21E-03	1.99E-07	1.21E-03
**Sphaero v Center**	NA	2.70E-01	7.34E-02	5.97E-02

Table S8: Acceleration (slope) and intercept fit to each velocity increase during three, one-second pulses. Numbers in parentheses denote 95% confidence intervals.

	Pulse	Acceleration (cm/s^2)	Intercept (cm/s)	R^2
**Serpenticone**	**1**	14.60 (14.20, 15.01)	1.416 (1.179, 1.653)	0.9527
	**2**	11.24 (10.74, 11.74)	-10.47 (-11.73, -9.204)	0.8865
	**3**	11.15 (10.44, 11.87)	-27.51 (-30.75, -24.27)	0.7901
**Oxycone**	**1**	17.46 (16.73, 18.19)	1.055 (0.6282, 1.482)	0.8987
	**2**	14.53 (13.65, 15.42)	-13.60 (-15.83, -11.36)	0.8074
	**3**	17.93 (15.63, 20.23)	-46.54 (-56.73, -36.36)	0.5528

Table S9: Peak angular velocity (about the vertical axis) during the yaw experiments. A one-way ANOVA with a Games-Howell post hoc test was used to distinguish the means of all trails between each morphotype.

	**Peak angular velocity (degrees/s)**			
**Trial**	**Sphaerocone**	**Center**	**Serpenticone**	**Oxycone**
**1**	433.68	296.46	155.39	89.99
**2**	408.79	292.41	160.45	87.28
**3**	484.63	255.48	153.79	88.23
**4**	429.01	268.26	153.88	83.63
**5**	457.06	274.27	148.57	93.34
**6**	432.07	285.47	152.42	86.06
**7**	431.62	247.98	148.02	89.60
**8**	446.25	274.91	154.95	91.38
**9**	436.81	268.68	153.58	94.52
**10**	446.97	274.44	152.50	89.71
**11**	454.24	303.27	145.94	92.99
**12**	432.23	260.47	136.25	85.79
**13**	447.03	288.34	149.28	92.36
**14**	462.34	304.41	163.93	95.00
**15**	469.66	291.52	141.00	90.35
**Average**	444.83	279.09	151.33	90.01
**SD**	18.88	17.21	6.96	3.36
	**ANOVA (p value)**		**% Difference of averages**	
**Sphaero v Center**	9.10E-15		45.79	
**Sphaero v Serp**	1.48E-14		98.46	
**Sphaero v Oxy**	1.44E-14		132.68	
**Center v Serp**	8.99E-15		59.37	
**Center v Oxy**	1.27E-14		102.45	
**Serp v Oxy**	5.11E-15		50.81	

Table S10: Moments of inertia (MOI) computed for each component of unique density (including some bulk density values; see methods) for the robots, virtual models representing living animals with theoretical morphologies, and two simple shapes ("Sphere" and "Disk"). The sphere has the same volume as the sphaerocone, and the disk has the same aspect ratio as the oxycone. All MOIs were computed in MeshLab. The simple shapes were calculated by hand for comparison.

Model	Serpenticone robot	Serpenticone virtual	Oxycone robot	Oxycone virtual	Sphaerocone robot	Sphaerocone virtual	Center robot	Center virtual	Sphere computed	Sphere calculated	Disk computed	Disk calculated
Material	MOI ($kg*m^2$)	MOI ($kg*m^2$)	MOI ($kg*m^2$)	MOI ($kg*m^2$)	MOI ($kg*m^2$)	MOI ($kg*m^2$)	MOI ($kg*m^2$)	MOI ($kg*m^2$)	MOI ($kg*m^2$)	MOI ($kg*m^2$)	MOI ($kg*m^2$)	MOI ($kg*m^2$)
PETG	1.304E-03		1.835E-03		8.568E-04		1.075E-03					
Electronics cartridge	1.059E-04		7.329E-05		1.966E-05		4.175E-05					
Bismuth counterweight	1.625E-04		1.991E-04		1.295E-04		9.033E-05					
Liquid	3.043E-05		3.896E-05		1.062E-05		2.139E-05					
Motor	4.779E-05	NA	6.376E-05	NA	1.185E-05	NA	3.141E-05	NA				
3.7V battery	2.719E-05		1.264E-05		6.712E-07		5.708E-06		NA	NA	NA	NA
7.4V battery	1.232E-04		1.001E-04		3.213E-05		5.537E-05					
Electronics	4.330E-05		3.054E-05		1.404E-05		1.662E-05					
Self-healing rubber	9.184E-09		5.700E-08		2.621E-08		1.969E-09					
Soft body		1.946E-03		2.047E-03		1.307E-03		1.447E-03				
Shell	NA	5.958E-04	NA	8.623E-04	NA	2.466E-04	NA	3.472E-04				
Water		NA		NA		NA		NA	1.491E-03	1.493E-03	2.057E-03	2.052E-03
**Total robot**	1.845E-03		2.354E-03		1.075E-03		1.338E-03		NA	NA	NA	NA
**Total virtual**	NA	2.542E-03	NA	2.909E-03	NA	1.553E-03	NA	1.794E-03				
% Difference	27.47		19.09		30.77		25.44		0.18		-0.23	

Table S11: Determination of the relative importance of hydrodynamic effects (drag, wake dynamics) and moment of inertia on rotational kinematics during yaw maneuverability experiments. The computed moments of inertia (Table S10) for the robots and hydrostatic models (representing the living animals) were used to compute angular velocity ω reached after one second of jetting at 0.3N (rows b and c, respectively). The observed ω values after jetting for one second (row d) are consistently about an order of magnitude lower, demonstrating that hydrodynamic effects dominate rotational kinematics. The hydrodynamic proportions of ω for the robot moment of inertia and "living animal" moment of inertia (rows e and f, respectively) were subtracted to determine the relative difference in these proportions (row g). This approach yields estimates of how much the lower moments of inertia in the robots influence rotational kinematics for each morphotype.

	Serpenticone	Oxycone	Sphaerocone	Center
(a) Lever arm (mm)	102.27	110.70	74.93	92.07
(b) Robot ω in vacuum (degrees/s)	952.95	808.47	1197.79	1182.97
(c) Animal ω in vacuum (degrees/s)	691.64	654.14	829.21	881.96
(d) Observed ω (degrees/s)	118.90 ± 6.32	78.35 ± 4.20	328.87 ± 18.92	270.54 ± 19.98
(e) Hydrodynamic proportion of ω (1-d/b)x100 (%)	87.52	90.31	72.54	77.13
(f) Hydrodynamic proportion of ω (1-d/c)x100 (%)	82.81	88.02	60.34	69.33
(g) Difference in proportions (e-f) (%)	4.71	2.29	12.20	7.81

Table S12: Standard deviations (in mm) of the tracking point distances for each robot and each type of experiment. Lateral-view tracking points were used during the horizontal movement experiments with either one or three jet pulses. The top-view tracking points were used for the rotation experiments about the vertical axis (yaw maneuverability).

Robot	Lateral-view tracking point distance(mm)	Top-view tracking point distance (mm)	Single pulse video 1	Single pulse video 2	Three pulses video 1	Three pulses video 2	Vertical rotation
Serpenticone	90.0	55.0	4.37	3.77	3.85	8.20	2.30
Oxycone	99.0	60.0	7.51	7.79	4.73	8.46	1.97
Sphaerocone	71.0	41.5	2.21	2.25	NA	NA	3.77
Center	83.5	38.0	1.17	2.22	NA	NA	4.16

Table S13: Shell and septum thicknesses and ratios of thickness and inner whorl height (from ventral shell of the current whorl to the ventral shell of the previous whorl). Errors represent standard deviations.

Whorl height (mm)	Shell Thickness (mm)	Septal Thickness (mm)	Shell Thickness / Whorl Height	Septal Thickness / Whorl Height
46.47	1.26	0.92	0.0271	0.0198
36.56	1.07	0.71	0.0293	0.0194
31.76	0.93	0.61	0.0293	0.0192
27.24	0.9	0.59	0.0330	0.0217
25.19	0.88	0.56	0.0349	0.0222
21.53	0.74	0.48	0.0344	0.0223
		Average:	0.0313 ± 0.0032	0.0207 ± 0.001

Table S14: Array instructions use to build each planispiral model. Multiple arrays were used in a piecewise manner to account for ontogenetic changes in coiling. These instructions were used in Blender (Blender Online Community) to replicate the adult whorl section while simultaneously translating, rotating, and scaling, creating a spiral.

Model	Array	Translation		Rotation	Scaling			Number of Replications	Aperture	
		y (mm)	z (mm)	x (degrees)	x	y	z		Length (mm)	Width (mm)
Sphaerocone	1	-0.3	-2.06	-1	0.99885	0.99845	0.9987	540	154.01619	202.93
	2	-0.3	-2.07	-1	0.99845	0.9981	0.9985	720		
	3	-0.31	-2.08	-1	0.9984	0.9979	0.9986	960		
Oxycone	1	-0.18	-1.86	-1	0.9977	0.9983	0.9977	2500	155.582	56.1466
Serpenticone	1	-0.101	-2.37	-1	0.9991	0.9994	0.9994	180	71.4243	75.1924
	2	-0.191	-2.52	-1	0.9995	0.9988	0.9988	181		
	3	-0.161	-2.44	-1	0.9994	0.999	0.9988	181		
	4	-0.141	-2.58	-1	0.9993	0.9992	0.9992	181		
	5	-0.251	-2.505	-1	0.9985	0.9984	0.9984	181		
	6	-0.225	-2.545	-1	0.9988	0.9986	0.9986	181		
	7	-0.225	-2.515	-1	0.9988	0.9986	0.9986	181		
	8	-0.225	-2.545	-1	0.9988	0.9986	0.9986	181		
	9	-0.223	-2.518	-1	0.9989	0.9986	0.9986	181		
	10	-0.284	-2.527	-1	0.9989	0.9982	0.9982	181		
	11	-0.3132	-2.524	-1	0.9983	0.998	0.998	181		
	12	-0.297	-2.524	-1	0.9986	0.9981	0.9982	1000		
Center	1	-0.0636	-0.803	-1	0.9992	0.9986	0.9988	3000	43.0605	42.8616

Table S15: Conch measurements in mm (corresponding to Fig. S13), and Westermann Morphospace parameters (*1*) for each planispiral model.

Model	a	a'	b	D	UD	w	U	Th
Oxycone	137.034	96.049	49.825	235.800	2.717	1.427	0.012	0.211
Serpenticone	46.204	41.647	49.175	213.224	125.373	1.109	0.588	0.231
Sphaerocone	77.311	59.913	101.497	150.000	12.776	1.290	0.085	0.677
Center	71.771	57.187	71.825	176.692	47.734	1.255	0.270	0.406
Nautilus	113.348	57.557	88.295	180.365	9.460	1.969	0.052	0.490

Table S16: Centers of each material of unique density (soft body, shell, cameral liquid, and cameral gas, and hydrostatic centers (buoyancy and mass). Note that only the y and z components are reported because these models are symmetrical.

	Soft body		Shell		Cameral liquid		Cameral gas		Center of buoyancy		Center of mass	
Model	y (mm)	z (mm)	y (mm)	z (mm)	y (mm)	z (mm)	y (mm)	z (mm)	y (mm)	z (mm)	y (mm)	z (mm)
Oxycone	-53.807	-31.013	-84.629	-23.472	-110.086	12.633	-110.086	12.633	-72.373	-18.971	-66.159	-27.268
Serpenticone	-83.250	-19.038	-90.569	-15.479	-104.230	-1.282	-104.230	-1.282	-87.730	-15.510	-85.353	-17.913
Sphaerocone	-54.446	-10.163	-61.636	-11.255	-71.842	-0.633	-71.842	-0.633	-56.965	-9.085	-55.719	-10.221
Center	-68.037	-15.720	-74.519	-12.695	-83.644	1.892	-83.644	1.892	-71.072	-12.641	-69.714	-14.678

Table S17: Volumes and masses of each model component. sb = soft body, sh = shell, cl = cameral liquid, cg = cameral gas, wd = water displaced, ct = camerae total, total = total center of mass.

Model	V_{sb} (cm^3)	V_{sh} (cm^3)	V_{cl} (cm^3)	V_{cg} (cm^3)	V_{wd} (cm^3)	V_{ct} (cm^3)	m_{sb} (g)	m_{sh} (g)	m_{cl} (g)	m_{cg} (g)	m_{wd} (g)	m_{total} (g)
Oxycone	594.153	141.295	23.496	223.014	981.957	246.510	623.267	358.888	24.083	0.269	1006.506	1006.506
Serpenticone	708.153	98.398	13.195	162.209	981.955	175.404	742.852	249.932	13.525	0.195	1006.504	1006.504
Sphaerocone	804.608	59.568	10.771	106.986	981.957	117.757	844.034	151.303	11.040	0.129	1006.506	1006.506
Center	745.191	78.537	24.544	133.686	981.958	158.230	781.705	199.483	25.157	0.161	1006.507	1006.507

Legend for Dataset S1

Theoretical planispiral models.

Available at https://doi.org/10.5281/zenodo.5684906

Description: Zipped folder containing virtual models (in .STL format) of theoretical ectocochleate cephalopods (oxycone, serpenticone, sphaerocone, and morphospace center). There are four components of each model used in hydrostatic calculations: 1) shell, 2) chambers (camerae), 3) soft body, and 4) water displaced. Each near-endmember morphotype has two versions: 1) Body chamber lengths allowing neutral buoyancy with *Nautilus*-like chamber liquid (~12%), and 2) Body chamber lengths of the representative specimens used to build the models (*Sphenodiscus* for oxycone, *Dactylioceras* for serpenticone, and *Goniatites* for sphaerocone).

Legend for Dataset S2

Digital models of biomimetic cephalopod robots.

Available at https://doi.org/10.5281/zenodo.6180801

Description: This dataset consists of a .zip folder containing: 1) the Arduino code uploaded to the robot microcontroller, 2) sample footage of the movement for each biomimetic robot during 3D motion tracking, and 3) 3D models in .stl format for each biomimetic robot (serpenticone, oxycone, sphaerocone, and morphospace center). Each robot morphotype has their own file including models of: both batteries (3.7V and 7.4V), counterweights cast with bismuth, the electronics cartridge, electronic components (microcontroller, charger/regulator, motor driver, wires, LED indicator, and IR sensor), the PETG impeller, chamber liquid and water pump liquid (LiquidALL.stl), a brushed DC motor, PETG parts 1-4, PETG lid, self-healing rubber valve, and the water displaced by the external model. The PETG parts were printed in natural colored PETG with solid infill and 0.12 mm vertical resolution.

SI References

1. K. A. Ritterbush, D. J. Bottjer, Westermann Morphospace displays ammonoid shell shape and hypothetical paleoecology. *Paleobiology*. **38** (2012), doi:10.1666/10027.1.
